# Finished Genome of the Fungal Wheat Pathogen *Mycosphaerella graminicola* Reveals Dispensome Structure, Chromosome Plasticity, and Stealth Pathogenesis

**DOI:** 10.1371/journal.pgen.1002070

**Published:** 2011-06-09

**Authors:** Stephen B. Goodwin, Sarrah Ben M'Barek, Braham Dhillon, Alexander H. J. Wittenberg, Charles F. Crane, James K. Hane, Andrew J. Foster, Theo A. J. Van der Lee, Jane Grimwood, Andrea Aerts, John Antoniw, Andy Bailey, Burt Bluhm, Judith Bowler, Jim Bristow, Ate van der Burgt, Blondy Canto-Canché, Alice C. L. Churchill, Laura Conde-Ferràez, Hans J. Cools, Pedro M. Coutinho, Michael Csukai, Paramvir Dehal, Pierre De Wit, Bruno Donzelli, Henri C. van de Geest, Roeland C. H. J. van Ham, Kim E. Hammond-Kosack, Bernard Henrissat, Andrzej Kilian, Adilson K. Kobayashi, Edda Koopmann, Yiannis Kourmpetis, Arnold Kuzniar, Erika Lindquist, Vincent Lombard, Chris Maliepaard, Natalia Martins, Rahim Mehrabi, Jan P. H. Nap, Alisa Ponomarenko, Jason J. Rudd, Asaf Salamov, Jeremy Schmutz, Henk J. Schouten, Harris Shapiro, Ioannis Stergiopoulos, Stefano F. F. Torriani, Hank Tu, Ronald P. de Vries, Cees Waalwijk, Sarah B. Ware, Ad Wiebenga, Lute-Harm Zwiers, Richard P. Oliver, Igor V. Grigoriev, Gert H. J. Kema

**Affiliations:** 1 USDA–Agricultural Research Service, Purdue University, West Lafayette, Indiana, United States of America; 2 Plant Research International B.V., Wageningen, The Netherlands; 3 Department of Botany and Plant Pathology, Purdue University, West Lafayette, Indiana, United States of America; 4 School of Veterinary and Biomedical Sciences, Murdoch University, Perth, Australia; 5 IBWF e.V., Institute for Biotechnology and Drug Research, Kaiserslautern, Germany; 6 HudsonAlpha Institute of Biotechnology, Huntsville, Alabama, United States of America; 7 DOE Joint Genome Institute, Walnut Creek, California, United States of America; 8 Rothamsted Research, Department of Plant Pathology and Microbiology, Harpenden, United Kingdom; 9 School of Biological Sciences, University of Bristol, Bristol, United Kingdom; 10 University of Arkansas, Fayetteville, Arkansas, United States of America; 11 Syngenta, Jealott's Hill Research Centre, Bracknell, United Kingdom; 12 Unidad de Biotecnología, Centro de Investigación Científica de Yucatán, A.C., (CICY), Mérida, México; 13 Department of Plant Pathology and Plant-Microbe Biology, Cornell University, Ithaca, New York, United States of America; 14 Architecture et Fonction des Macromolecules Biologiques, CNRS, Marseille, France; 15 Wageningen University and Research Centre, Wageningen, The Netherlands; 16 USDA–Agricultural Research Service, Ithaca, New York, United States of America; 17 Diversity Arrays Technology Pty Ltd, Yarralumla, Australia; 18 Embrapa Meio-Norte, Teresina, Piauí, Brazil; 19 Bayer CropScience AG, Monheim, Germany; 20 Embrapa-Cenargen, Brasilia, Brazil; 21 Department of Genetics, Seed and Plant Improvement Institute, Karaj, Iran; 22 Plant Pathology, Institute of Integrative Biology, Swiss Federal Institute of Technology (ETH), Zürich, Switzerland; 23 CBS–KNAW Fungal Biodiversity Centre, Utrecht, The Netherlands; 24 Environment and Agriculture, Curtin University, Bentley, Australia; Fred Hutchinson Cancer Research Center, United States of America

## Abstract

The plant-pathogenic fungus *Mycosphaerella graminicola* (asexual stage: *Septoria tritici*) causes septoria tritici blotch, a disease that greatly reduces the yield and quality of wheat. This disease is economically important in most wheat-growing areas worldwide and threatens global food production. Control of the disease has been hampered by a limited understanding of the genetic and biochemical bases of pathogenicity, including mechanisms of infection and of resistance in the host. Unlike most other plant pathogens, *M. graminicola* has a long latent period during which it evades host defenses. Although this type of stealth pathogenicity occurs commonly in Mycosphaerella and other Dothideomycetes, the largest class of plant-pathogenic fungi, its genetic basis is not known. To address this problem, the genome of *M. graminicola* was sequenced completely. The finished genome contains 21 chromosomes, eight of which could be lost with no visible effect on the fungus and thus are dispensable. This eight-chromosome dispensome is dynamic in field and progeny isolates, is different from the core genome in gene and repeat content, and appears to have originated by ancient horizontal transfer from an unknown donor. Synteny plots of the *M. graminicola* chromosomes versus those of the only other sequenced Dothideomycete, *Stagonospora nodorum*, revealed conservation of gene content but not order or orientation, suggesting a high rate of intra-chromosomal rearrangement in one or both species. This observed “mesosynteny” is very different from synteny seen between other organisms. A surprising feature of the *M. graminicola* genome compared to other sequenced plant pathogens was that it contained very few genes for enzymes that break down plant cell walls, which was more similar to endophytes than to pathogens. The stealth pathogenesis of *M. graminicola* probably involves degradation of proteins rather than carbohydrates to evade host defenses during the biotrophic stage of infection and may have evolved from endophytic ancestors.

## Author Summary

The plant-pathogenic fungus *Mycosphaerella graminicola* causes septoria tritici blotch, one of the most economically important diseases of wheat worldwide and a potential threat to global food production. Unlike most other plant pathogens, *M. graminicola* has a long latent period during which it seems able to evade host defenses, and its genome appears to be unstable with many chromosomes that can change size or be lost during sexual reproduction. To understand its unusual mechanism of pathogenicity and high genomic plasticity, the genome of *M. graminicola* was sequenced more completely than that of any other filamentous fungus. The finished sequence contains 21 chromosomes, eight of which were different from those in the core genome and appear to have originated by ancient horizontal transfer from an unknown donor. The dispensable chromosomes collectively comprise the dispensome and showed extreme plasticity during sexual reproduction. A surprising feature of the *M. graminicola* genome was a low number of genes for enzymes that break down plant cell walls; this may represent an evolutionary response to evade detection by plant defense mechanisms. The stealth pathogenicity of *M. graminicola* may involve degradation of proteins rather than carbohydrates and could have evolved from an endophytic ancestor.

## Introduction

The ascomycete fungus *Mycosphaerella graminicola* (Figure S1) causes septoria tritici blotch (STB), a foliar disease of wheat that poses a significant threat to global food production. Losses to STB can reduce yields of wheat by 30 to 50% with a huge economic impact [1]; global expenditures for fungicides to manage STB total hundreds of millions of dollars each year [2]–[3]. This fungus is difficult to control because populations contain extremely high levels of genetic variability [4] and it has very unusual biology for a pathogen. Unlike most other plant pathogens [5]–[7], *M. graminicola* infects through stomata rather than by direct penetration and there is a long latent period of up to two weeks following infection before symptoms develop. The fungus evades host defenses [8] during the latent phase, followed by a rapid switch to necrotrophy immediately prior to symptom expression 12–20 days after penetration [5], [9]–[10]. Such a switch from biotrophic to necrotrophic growth at the end of a long latent period is an unusual characteristic shared by most fungi in the genus Mycosphaerella. Very little is known about the cause or mechanism of this lifestyle switch [9]–[10] even though Mycosphaerella is one of the largest and most economically important genera of plant-pathogenic fungi.

A striking aspect of *M. graminicola* genetics is the presence of many dispensable chromosomes [11]. These can be lost readily in sexual progeny with no apparent effect on fitness. However, the structure and function of dispensable chromosomes are not known. Here we report the first genome of a filamentous fungus to be finished according to current standards [12]. The 21-chromosome, 39.7-Mb genome of *M. graminicola* revealed an apparently novel origin for dispensable chromosomes by horizontal transfer followed by extensive recombination, a possible mechanism of stealth pathogenicity and exciting new aspects of genome structure. The genome provides a finished reference for the Dothideomycetes, the largest class of ascomycete fungi, which also includes the apple scab pathogen *Venturia inaequalis*, the southern corn leaf blight pathogen *Cochliobolus heterostrophus*, the black Sigatoka pathogen of banana, *M. fijiensis*, and numerous other pathogens of almost every crop.

## Results

### Features of the finished genome

**Figure 1:**
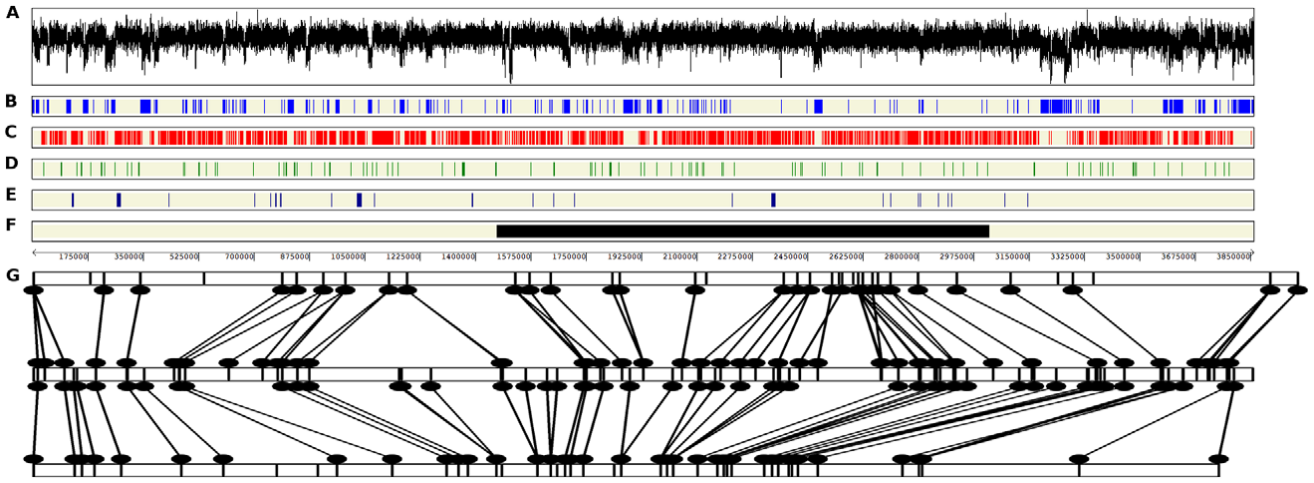
Features of chromosome 2 of *Mycosphaerella graminicola* and alignment to genetic linkage maps. A, Plot of GC content. Areas of low GC usually correspond to regions of repetitive DNA. B, Repetitive regions of the *M. graminicola* genome. C, Single-copy (red) regions of the *M. graminicola* genome. D, Locations of genes for proteins containing signal peptides. E, Locations of homologs involved in pathogenicity or virulence that have been experimentally verified in species pathogenic to plant, animal or human hosts. F, Approximate locations of quantitative trait loci (QTL) for pathogenicity to wheat. G, Alignments between the genomic sequence and two genetic linkage maps of crosses involving isolate IPO323. Top half, Genetic linkage map of the cross between IPO323 and the Algerian durum wheat isolate IPO95052. Bottom half, Genetic linkage map of the cross between bread wheat isolates IPO323 and IPO94269. The physical map represented by the genomic sequence is in the center. Lines connect mapped genetic markers in each linkage map to their corresponding locations on the physical map based on the sequences of the marker loci. Exceptions to the almost perfect alignment between the three maps are indicated by crossed lines, most likely due to occasional incorrect scorings of the marker alleles. Chromosome 2 was used for this illustration because no QTL mapped to chromosome 1.

The finished genome of *M. graminicola* isolate IPO323 consists of 21 complete chromosomes, telomere to telomere (Figure S2), with the exceptions of one telomere of chromosome 21 and two internal gaps of unclonable DNA that are missing from chromosome 18 (Table 1). Alignments between the 21 chromosomes and two genetic linkage maps yielded an excellent correspondence (Figure 1 and Figure S3), representing the most complete and the first finished sequence of a filamentous fungus. The next most complete genome of a filamentous fungus is that of *Aspergillus fumigatus*, which did not include centromere sequences and contained 11 gaps in total [13]. The complete 43,960-bp mitochondrial genome also was obtained and has been described elsewhere [14].

**Table 1:** Sizes and gene contents of the 21 chromosomes of *Mycosphaerella graminicola* isolate IPO323.

Chromosome		All genes		Unique genes^a^		Signal peptides	Average gene size (bp)	Genes/Mb DNA	Percent G+C	Percent repetitive	milRNAs/Mb DNA^b^
Number	Size	Number	Annotated	Number	Annotated						
1	6,088,797	1,980	1,258	1,067	497	208	1338.6	325	53.1	9.5	9.7
2	3,860,111	1,136	650	607	238	108	1402.7	294	52.4	15.7	9.6
3	3,505,381	1,071	630	583	246	122	1337.1	306	52.6	14.2	6.3
4	2,880,011	821	498	421	182	81	1388.6	285	52.2	16.1	13.2
5	2,861,803	778	489	389	180	91	1352.6	272	52.0	19.1	18.9
6	2,674,951	692	427	328	152	66	1353.0	259	51.4	22.2	12.3
7	2,665,280	766	357	462	131	96	1202.7	287	52.6	14.0	16.1
8	2,443,572	689	397	384	159	62	1311.2	282	51.7	17.6	13.5
9	2,142,475	604	353	305	134	69	1345.1	282	51.5	20.8	18.7
10	1,682,575	516	298	266	110	46	1418.7	307	52.5	14.1	9.5
11	1,624,292	488	279	270	115	65	1352.5	300	52.8	10.5	5.5
12	1,462,624	408	227	232	96	59	1254.3	279	52.3	14.5	10.9
13	1,185,774	330	183	165	68	47	1195.7	278	52.0	17.8	17.7
14	773,098	114	25	48	5	3	920.1	147	48.5	36.7	23.3
15	639,501	86	6	44	1	2	773.7	134	51.0	34.4	25.0
16	607,044	88	5	40	1	5	898.5	145	51.5	25.6	31.3
17	584,099	78	6	36	1	1	777.9	134	52.0	26.4	18.8
18^c^	573,698	64	7	28	4	0	965.1	112	48.6	40.3	33.1
19	549,847	87	8	53	3	4	658.3	158	51.3	25.1	23.6
20	472,105	79	4	41	2	4	863.1	167	51.5	21.1	25.4
21^d^	409,213	58	4	21	1	2	921.6	142	51.9	30.1	14.7
Total	39,686,251	10,933	6,111	5,790	2,326	1,141					13.5

a At a BLAST cutoff value of 1×e^−20^.

b Predicted numbers of loci for pre-microRNA-like small RNAs.

c This chromosome contains two internal gaps of unclonable DNA marked by gaps of 1.4 and 4.5 kb; all other chromosomes are complete.

d The sequence of one telomere is missing from this chromosome; all other telomeres are complete.

### Sexual activation of chromosome plasticity and repeat-induced point mutation

**Figure 2:**
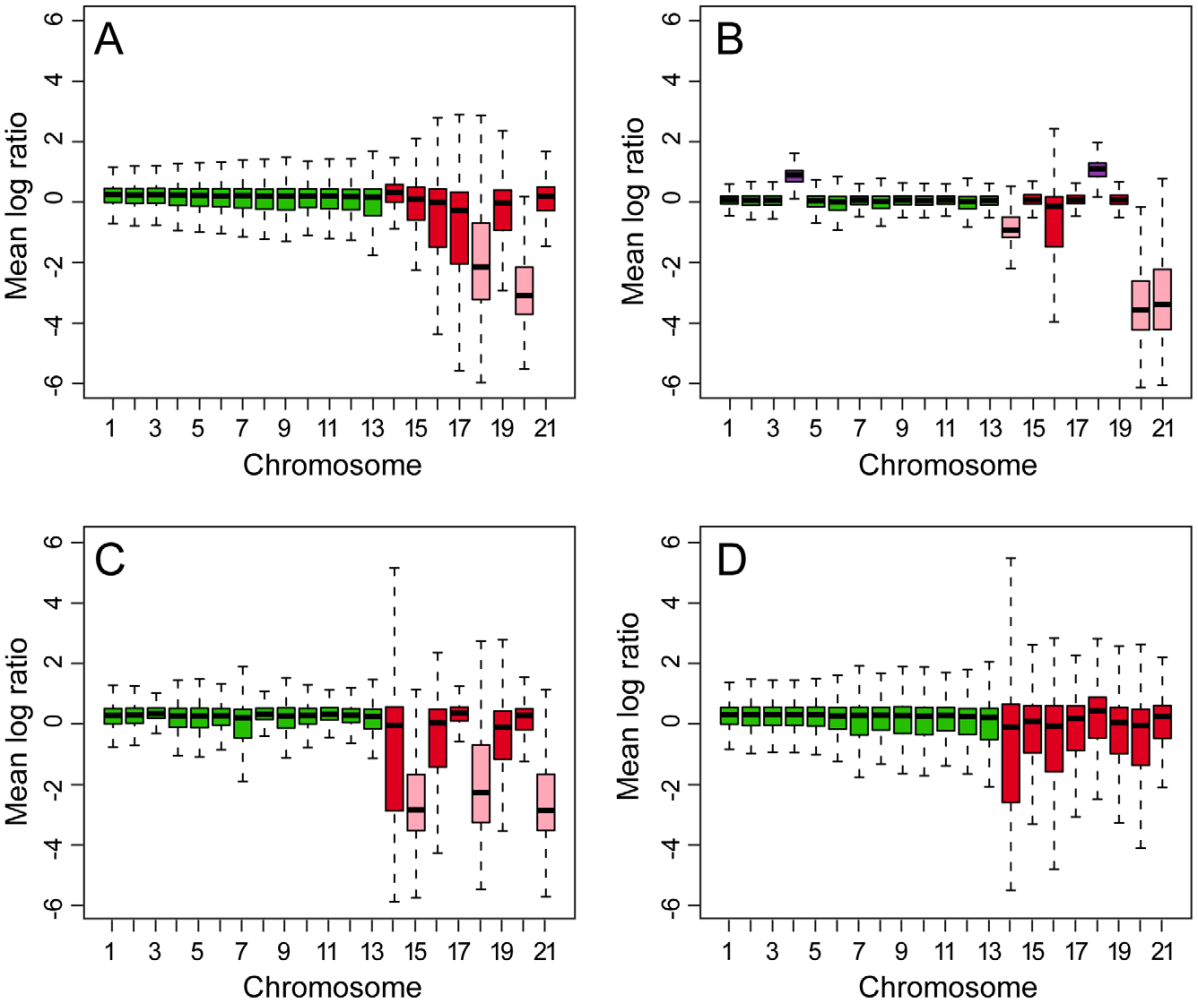
Box plots of comparative genome hybridizations (CGH) of DNA from five isolates of *Mycosphaerella graminicola* to a whole-genome tiling array made from the finished sequence of isolate IPO323. A, CGH between IPO323 and the Dutch field isolate IPO94269. B, CGH between IPO323 and progeny isolate #51 from the cross between IPO323 and IPO94269. C, CGH between IPO323 and progeny isolate #2133 of the cross between IPO323 and IPO95052. D, CGH between IPO323 and Algerian field isolate IPO95052, which was isolated from and is adapted to durum (tetraploid) wheat. The genomic difference between the strains for each CGH is shown by 21 box plots, one for each chromosome of *M. graminicola*. The horizontal line in each box is the median log ratio of hybridization signals of the two strains; the upper and lower ends of a box represent the 25% and 75% quartiles. The whiskers extending from each box indicate 1.5 times the interquartile range, the distance between the 25% and 75% quartiles. The larger the deviation from 0, the greater the difference between the strains for a particular chromosome. Pink boxes that are significantly less than the zero line indicate missing chromosomes. The purple boxes in panel B (4 and 18) that are significantly higher than the zero line indicate chromosomes that are disomic.

Comparative genome hybridizations using a whole-genome tiling array made from the genome sequence of IPO323 demonstrated striking sexually activated chromosomal plasticity in progeny isolates (Figure 2) and chromosome number polymorphisms in field isolates. For example, isolate IPO94269, a field strain from bread wheat in the Netherlands, was missing two chromosomes that were present in IPO323 (Figure 2A).

Sexual-driven genome plasticity was particularly evident among progeny isolates in the two mapping populations, including losses of chromosomes that were present in both parents and disomy for others [11]. For example, progeny isolate #51 of the cross between IPO323 and IPO94269 lost chromosomes 14 and 21 (Figure 2B) even though they were present in both parents. This isolate also was missing chromosome 20, which was polymorphic for presence between the parents of the cross. More surprisingly, this isolate was disomic for chromosomes 4 and 18 (Figure 2B), indicating that chromosomes can be both gained and lost during meiosis. For chromosome 18, both copies must have originated from IPO323 because no homolog was present in IPO94269. Molecular markers for chromosome 4 appeared to be heterozygous indicating that both parents contributed a copy to progeny isolate #51 (data not shown). Progeny isolate #2133 of the cross between isolates IPO323 and IPO95052 showed loss of three dispensable chromosomes (15, 18 and 21) that were present in both parents (Figure 2C), most likely due to non-disjunction during meiosis. Thus, extreme genome plasticity was manifested as chromosome number and size polymorphisms [11] that were generated during meiosis and extended to core as well as dispensable chromosomes.

The whole-genome hybridizations also indicated that the core and dispensable chromosomes can be remarkably uniform for gene content, given the high capacity of the latter for change. Comparative genome hybridizations between IPO323 and IPO95052, an isolate from a field of durum wheat in Algeria, showed that they had the same complement of core and dispensable chromosomes (Figure 2D). This was surprising, because populations of the pathogen from durum wheat (a tetraploid) usually are adapted to that host and not to hexaploid bread wheat, yet the chromosomal complements of isolates from these hosts on different continents were the same.

Evidence for repeat-induced point mutation (RIP), a mechanism in fungi that inactivates transposons by introducing C to T transitions in repeated sequences [15]–[16], was seen in genome-wide analyses of transition∶transversion ratios in long terminal repeat (LTR) pairs from 20 retrotransposon insertions which had 255 transitions and 6 transversions for a ratio of 42.5∶1. Similarly high transition∶transversion ratios were found in all repetitive sequences analyzed and extended to the coding regions in addition to the LTRs [17]. The reverse transcriptase coding regions from transposon families RT11 and RT15 had transition∶transversion ratios of 27.8∶1 and 25.3∶1, respectively, instead of the 1∶1 ratio expected among 6,939 mutations analyzed. This high incidence of transitions most likely reflects changes caused by RIP. The coding regions of all transposons with more than 10 copies included stop codons that prevent proper translation, indicating that they were inactivated.

### Core and dispensable chromosomes are highly divergent

There were significant differences in structure and gene content between the 13 core and eight dispensable chromosomes (Table 1 and Table 2); the latter are referred to collectively as the dispensome. The dispensome constituted about 12% of the genomic DNA but contained only 6% of the genes. In contrast, the 13 core chromosomes had twice as many genes per Mb of DNA, about half as much repetitive DNA, a significantly higher G+C content, and much higher numbers of unique genes (Table 1 and Table 2). Genes in the dispensome were significantly shorter, usually were truncated relative to those on the core chromosomes (Table 2) and had dramatic differences in codon usage (Figure S4).

**Table 2:** Differences between essential and dispensable chromosomes in the genome of *Mycosphaerella graminicola* isolate IPO323.

	Chromosomes		
Statistic	Core (1–13)	Dispensable (14–21)	Combined (1–21)
Size in bp			
Total	35,077,646	4,608,605	39,686,251
Mean	2,698,280	576,076***	1,889,821
Percent	88.4	11.6	100.0
All genes			
Total	10,279	654	10,933
Mean	790.7	81.8***	521
Percent of total	94.0	6.0	100.0
Unique genes^a^			
Total	5,479	311	5,790
Mean	421.5	38.9***	276
Percent of all	53.3	47.6	53.0
Annotated genes			
Total	6,046	65	6,111
Mean	465.1	8.1***	291.0
Percent of all	58.8	9.9	55.9
Unique total	2,308	18	2,326
Unique mean	177.5	2.3***	110.8
Transcript size, mean in bp	1327.1	847.3***	1144.3
Gene density, *Mb^−1^*	288.9	142.4***	233.1
Repetitive DNA, mean	15.9%	30.0%***	21.2%
G+C, mean	52.3%	50.9%**	51.7%

a At a BLAST cutoff value of 1×e^−20^.

***The mean for the dispensable chromosomes is significantly different from that for the essential chromosomes at P<0.001 by one-tailed t test.

**The mean for the dispensable chromosomes is significantly different from that for the essential chromosomes at P = 0.012 by one-tailed t test.

**Figure 3:**
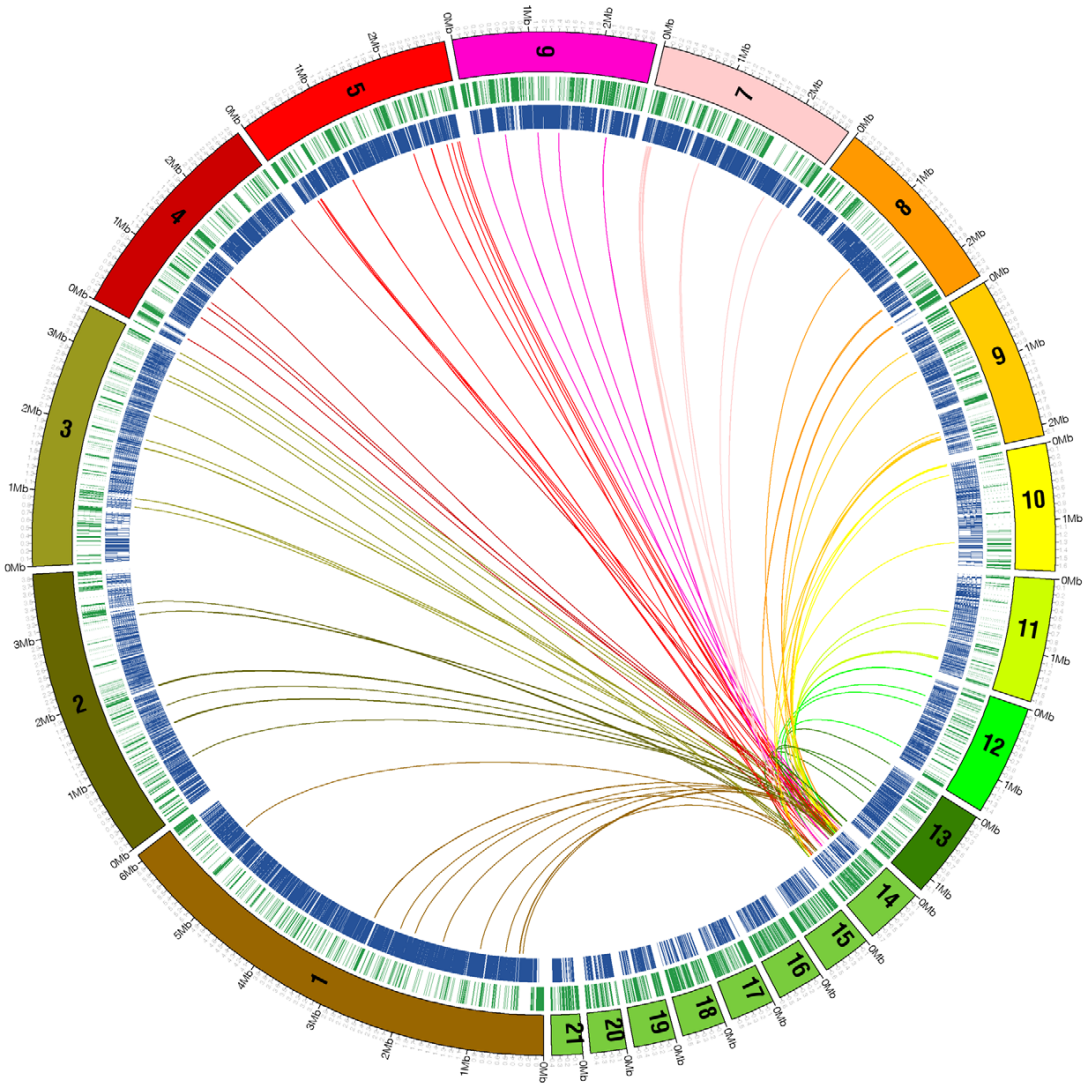
Analysis of genes that are shared between dispensable chromosome 14 and the 13 core chromosomes of *Mycosphaerella graminicola* isolate IPO323. Each chromosome is drawn to scale as a numbered bar around the outer edge of the circle, and the sequence was masked for repetitive DNA prior to analysis. Lines connect regions of 100 bp or larger that are similar between each core chromosome and the corresponding region on chromosome 14 at 1×e^−5^ or lower. Chromosome 14 is an amalgamation of genes from all of the core chromosomes but they are mixed together with no synteny. Genes on the other dispensable chromosomes were not included in this analysis.

About 59% of the genes on core chromosomes could be annotated compared to only 10% of those on the dispensome (Table 2). Some unique genes in the dispensome with intact, presumably functional reading frames, had possible paralogs on the core chromosomes (Figure S5) that appeared to be inactivated by mutations (Figure S6). A majority of the annotated dispensome genes coded for putative transcription factors or otherwise may function in gene regulation or signal transduction (Table S1). Most of the redundant genes on the dispensome were copies of genes present on core chromosomes, yet no syntenic relationships could be identified. Instead, each dispensable chromosome contained genes and repetitive sequences from all or most of the core chromosomes (Figure 3 and Figure S7) with additional unique genes of unknown origin. Sharing of genetic material applied to core chromosomes as well as the dispensome, consistent with a high level of recombination (Figure S8). Whether the primary direction of transfer is from core to dispensable chromosomes or vice versa is not known.

The dispensome contained fewer genes encoding secreted proteins such as effectors and other possible pathogenicity factors compared to the core set. Signal peptides showed no enrichment on the dispensome (Table S1) except for a few clusters overlapping with transposon-related repeats. Although mature microRNAs have not been demonstrated in fungi, they may be important regulatory molecules. In the *M. graminicola* genome, 418 non-overlapping loci potentially encoding pre-microRNA-like small RNA (pre-milRNA) were predicted computationally based on the RFAM database [18]. This number was similar to the 434 loci predicted in the 41-Mb genome of *Neurospora crassa* using the same approach. Of the 418 putative pre-milRNA loci predicted in the genome of *M. graminicola*, 88 (21%) are located on the 11% of the genome present as dispensome. This is about twice as much as is expected on the basis of a random distribution. Therefore, the dispensome is enriched for pre-milRNA loci.

The 418 pre-milRNA loci code for 385 non-redundant pre-milRNA sequences that can give rise to distinguishable mature milRNAs. The occurrence of mature milRNAs derived from the predicted set was analyzed in a small-RNA data consisting of almost 6 million reads (Illumina platform) generated from germinated spores of *M. graminicola* isolate IPO323 (Table S2).

Many of the non-redundant predicted milRNA sequences were represented in the RNA reads, at widely different amounts per sequence. In total, 65 of the 385 non-redundant sequences were observed 10 times or more. Two predicted sequences occurred more than a thousand times each, experimentally confirming the presence of putatively mature milRNAs derived from computationally predicted pre-milRNA sequences. In *N. crassa*, computationally predicted putative milRNA sequences also were confirmed experimentally [19], supporting the likelihood of their existence in *M. graminicola*.

The origin of the dispensome of *M. graminicola* is not clear. The two most likely origins would be degeneration of copies of the core chromosomes or by horizontal transfer. Disomy for core chromosomes, as seen in one of the progeny isolates, could provide the origin for a dispensable chromosome. If one of the two chromosome copies became preferentially subject to RIP followed by breakage or interstitial deletions this could result in a degenerated copy of that core chromosome. However, in that case we would expect the dispensome to share large regions of synteny with specific core chromosomes, and this was not observed, which renders this explanation less likely.

The large differences in codon usage between core and dispensable chromosomes could be explained by horizontal transfer or possibly by RIP. To discriminate between these hypotheses, RIP was simulated on the genes of the core chromosomes. Principal components analysis (PCA) of the simulated data set did not reduce the differences in codon bias (Figure S9A); if anything, it made them farther apart. This result was consistent whether it included only putative functional, truncated copies or entire pseudogenes after RIPping (data not shown). DeRIPping of genes on the dispensable chromosomes also did not affect the results (Figure S9B), so RIP could not explain the observed differences in codon usage between core and dispensable chromosomes. PCA of a sample of genes shared between core and dispensable chromosomes showed few differences in codon bias (Figure S9C) or amino acid composition (Figure S9D), consistent with an origin by duplication and exchange among chromosomes. This conclusion was supported when the analysis was expanded to include all genes with putative homologs on core and dispensable chromosomes (Figure S9E) even though these genes had a very different codon usage compared to the entire sets of genes on the core chromosomes (Figure S9F).

To test the horizontal transfer hypothesis, additional PCAs were performed on simulated horizontal transfer data sets made by combining the genome of *M. graminicola* with those of two other fungi. Best non-self BLAST hits for genes on the *M. graminicola* dispensome most often were to fungi in the Pleosporales or Eurotiales (Table S3), so published genomes from species representing those orders were chosen for analysis. PCA of the combined genomes of *M. graminicola* and *Stagonospora nodorum* (representing the Pleosporales) gave separate, tight clusters for the core chromosomes of *M. graminicola* versus most of those from *S. nodorum* (Figure S10A). Dispensable chromosomes of *M. graminicola* formed a looser, distinct cluster, and a fourth cluster was comprised of *M. graminicola* chromosome 14 plus scaffolds 44 and 45 of *S. nodorum* (Figure S10A); this may indicate the existence of dispensable chromosomes in the latter species. Analysis of the combined genomes of *M. graminicola* plus *Aspergillus fumigatus* (Eurotiales) gave a similar result (Figure S10B). The separate clustering by PCA of the *M. graminicola* dispensome and core chromosomes is consistent with an origin by horizontal transfer, but not from either of the two species tested. PCA on the frequencies of repetitive elements also indicated a separation between core and dispensable chromosomes (Figure S11), consistent with the horizontal transfer hypothesis.

**Figure 4:**
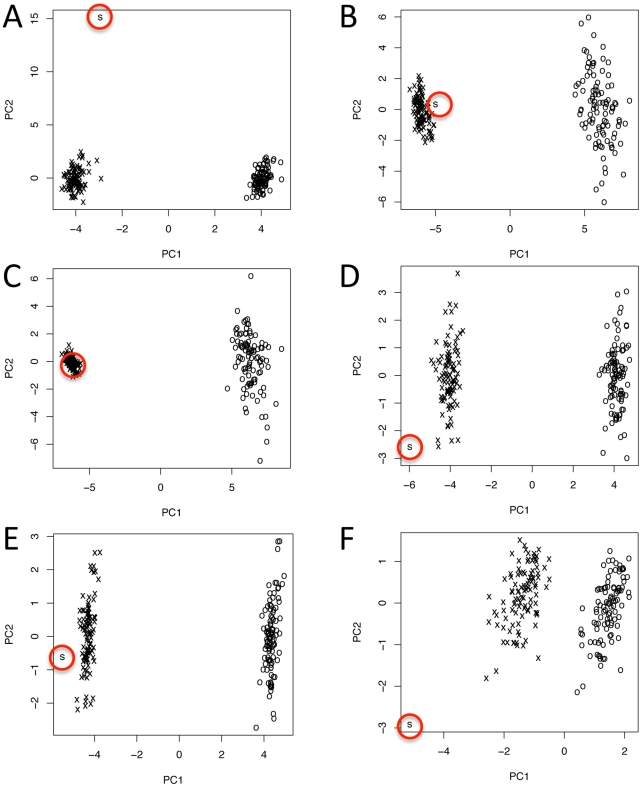
Principal Component Analysis of: S, observed genes on the dispensome; O, observed samples of genes on the core chromosomes before mutation; and x, samples of genes from the core chromosomes after mutation. Mutation was simulated using observed frequencies of all mutations in families of transposable elements with ten or more copies, and included mutations from RIP and other processes. Mutating the samples of genes from the core chromosomes always made them more similar to the observed value for the dispensome but only rarely included the dispensome value (see panel C). This occurred primarily with codon preference and GC content by amino acid, which are the quantities that are least subject to natural selection for protein function. A, amino acid frequency using the values for the aligned sequence with the highest GC content to build the table of mutation frequencies; B, codon preference using the consensus of the aligned sequences to make the table of mutation frequencies covering only the 5′ portion of each gene; C, codon preference using the values for the aligned sequence with the highest GC content to build the table of mutation frequencies covering only the 5′ portion of each gene; D, codon usage using the values for the aligned sequence with the highest GC content to build the table of mutation frequencies but with all mutation frequencies cut in half; E, codon usage using the values for the aligned sequence with the highest GC content to build the table of mutation frequencies; and F, GC skew using the consensus of the aligned sequences to make the table of mutation frequencies. The first principal component always separated out the pre- and post-mutated chromosome samples. The locations of the observed values for the dispensome (S) are circled.

A more refined test of the RIP hypothesis was performed by using the observed rates of all mutations in families of transposons with 10 or more elements to simulate mutational changes on replicated samples drawn from the core chromosomes. Observed mutation rates were calculated from aligned sequences; multicopy transposons were chosen for this analysis because they are the most likely to have been processed through the RIP machinery so will reflect the actual biases that occur in *M. graminicola*. Codon bias and other parameters in the mutated samples were then compared to those in the dispensome and in the original, non-mutated samples. Application of the mutational changes moved the samples drawn from the core chromosomes closer to the value observed for the dispensome, but the dispensome remained distinct except for a few of the analyses that are least likely to be affected by selection (Figure 4). This confirmed that the dispensome has been subject to RIP but that this alone was not sufficient to explain the observed pattern of codon usage.

### A new type of synteny

**Figure 5:**
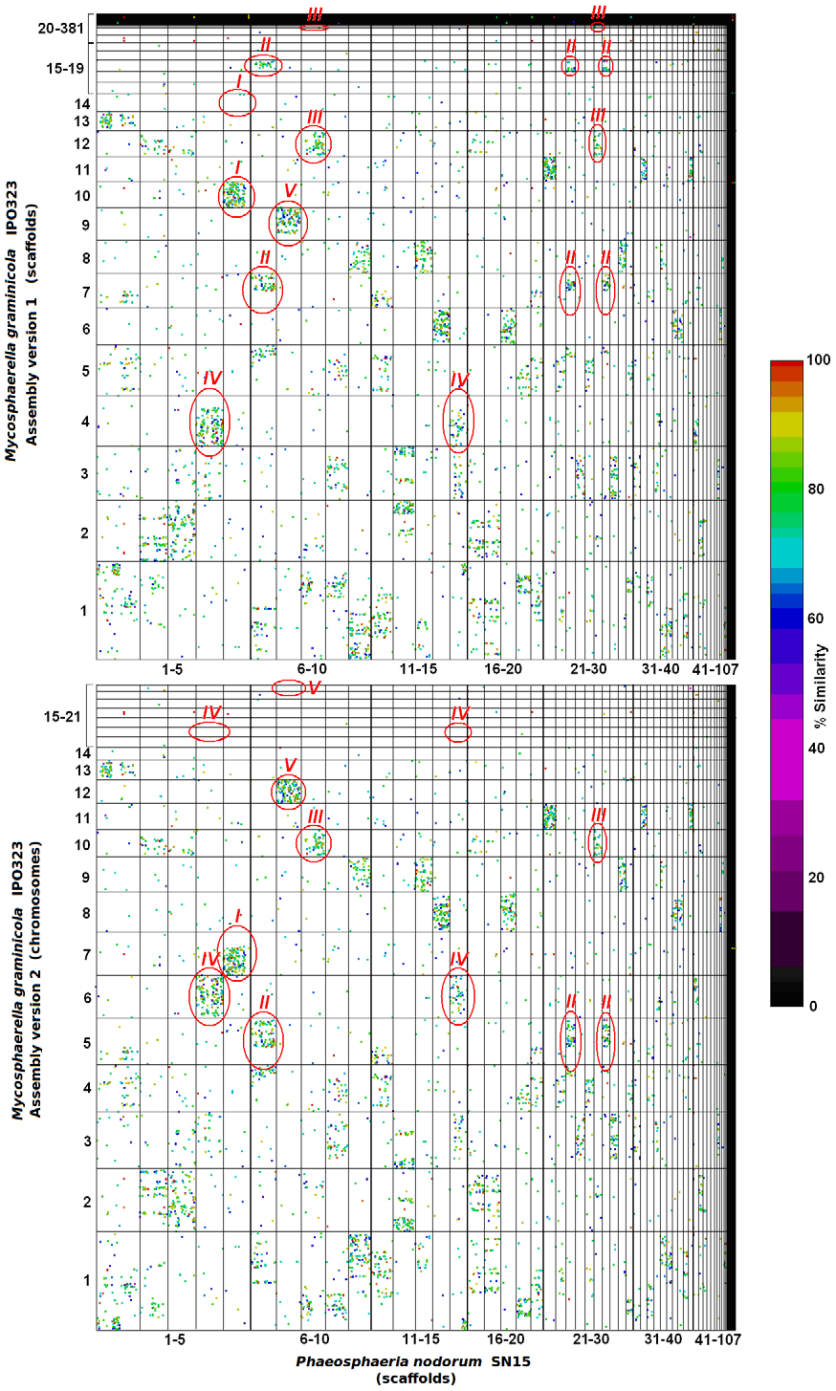
Comparisons of *Mycosphaerella graminicola* genome assembly versions 1 and 2 against that of *Stagonospora nodorum* isolate SN15. Scaffolds/chromosomes are ordered along their respective axes according to both decreasing length and increasing number. The 6-frame translations of both genomes were compared via MUMMER 3.0 [53]. Homologous regions are plotted as dots, which are color coded for percent similarity as per the bar on the right. Amendments made in the version 2 assembly and their corresponding regions in assembly version 1 are circled in red. Version 2 chromosomes 5 (B, circle II), 7 (B, circle I) and 10 (B, circle III) were derived from joined version 1 scaffolds 7 and 17 (A, circle II), 10 and 14 (A, circle I) and 12 and 22 (A, circle III), respectively, validating the method. Observation of the mesosyntenic pattern also could be used to identify inappropriately joined scaffolds. For example, *M. graminicola* v2 chromosomes 6 and 16 (B, circle IV) and 12 and 21 (B, circle V) were derived from split version 1 scaffolds 4 (A, circle IV) and 9 (A, circle V), respectively. These scaffolds are characterized by an abrupt termination of the mesosyntenic block at the split point as indicated by red lines (A, circles IV and V). A total of 21 predictions was made and 14 were validated.

Pairwise sequence comparisons between the chromosomes of *M. graminicola* and scaffolds of *Stagonospora nodorum*, another wheat pathogen in the Dothideomycetes but in a different order from Mycosphaerella, revealed multiple regions with approximately 70–90% similarity (Figure 5). However, the similarity did not extend to the dispensome, which generally was different from all of the *S. nodorum* scaffolds. Detailed examination showed that each region of similarity generally represents only one or a few genes in both organisms. Comparisons between the initial draft genome (version 1.0) of *M. graminicola* (Figure 5A) and the finished sequence (Figure 5B) revealed some misassemblies and also indicated scaffolds that ultimately were joined in the final assembly.

A surprising result was that the dot-plot patterns were very different from those that characterize the macro- or microsynteny seen in other organisms when viewed at a whole-scaffold/chromosome scale. Instead of the expected diagonal lines indicating chromosomal regions with content in the same order and orientation, the dots are scattered quasi-randomly within ‘blocks’ defined by scaffold/chromosome boundaries (Figure 5). For many *S. nodorum* scaffolds the vast majority of dots related are shared exclusively with one or a small number of *M. graminicola* chromosomes. For example, there are predominant one-to-one relationships between *M. graminicola* version 3 chromosomes 11 and 12 with *S. nodorum* scaffolds 21 and 7 (Figure 5B, circle V), respectively. Similarly, *M. graminicola* chromosomes 5–10 each had strong relationships with 2 to 4 scaffolds of *S. nodorum*. We refer to this conservation of gene content but not order or orientation among chromosomes as ‘mesosynteny’. Analyses of additional genomes has shown that mesosynteny as defined here occurs among all Dothideomycetes tested and may be unique to that class of fungi (data not presented).

### Mesosynteny as a tool to assist genome assembly

Macrosyntenic relationships are used commonly to assist the assembly and finishing of fragmented genome sequences [20]–[23], particularly in prokaryotes. Sequences that are macrosyntenic to a long segment of a closely related genome are highly likely to be joined physically. If mesosynteny between a new genome assembly and a reference genome also may be used to suggest scaffolds that should be juxtaposed it could significantly reduce the cost and complexity of assembling and finishing genomes. To test whether mesosynteny could be used to predict scaffold or contig joins in a genomic sequence, versions 1 and 2 of the *M. graminicola* genome assembly were analyzed to determine whether any of the improvements in the finished genome could have been predicted bioinformatically by mesosynteny (Dataset S1).

The first version of the *M. graminicola* genome consisted of 129 scaffolds (http://genome.jgi-psf.org/Mycgr1/Mycgr1.home.html). Comparison of *M. graminicola* version 1 scaffolds with those of the *P. nodorum* genome predicted all scaffold joins made in version 2 (Figure 5, Dataset S1). Version 1 scaffolds 10 and 14 (Figure 5: group I), 7 and 17 (groups II, VII and IX), and 12 and 22 (groups III and VIII) were joined into chromosomes 7, 5 and 10, respectively. Mesosynteny also indicated both instances where version 1 scaffolds were assembled incorrectly and subsequently were split in version 2. Compared to the scaffolds of *P. nodorum*, *M. graminicola* version 1 scaffold 4 exhibited regions of mesosynteny adjacent to regions of no synteny. Corrections to the assembly made in version 2 separated these two distinct regions into separate chromosomes. Version 1 scaffolds 4 and 9 (Figure 5: groups IV/VI and V) were corrected to version 2 chromosomes 6 and 16 (Figure 5: group IV/VI) and chromosomes 12 and 21 (Figure 5: group V) respectively. Mesosynteny was remarkably successful and has great potential to assist the assembly and finishing of fungal genomes.

### A mechanism of stealth pathogenesis

**Figure 6:**
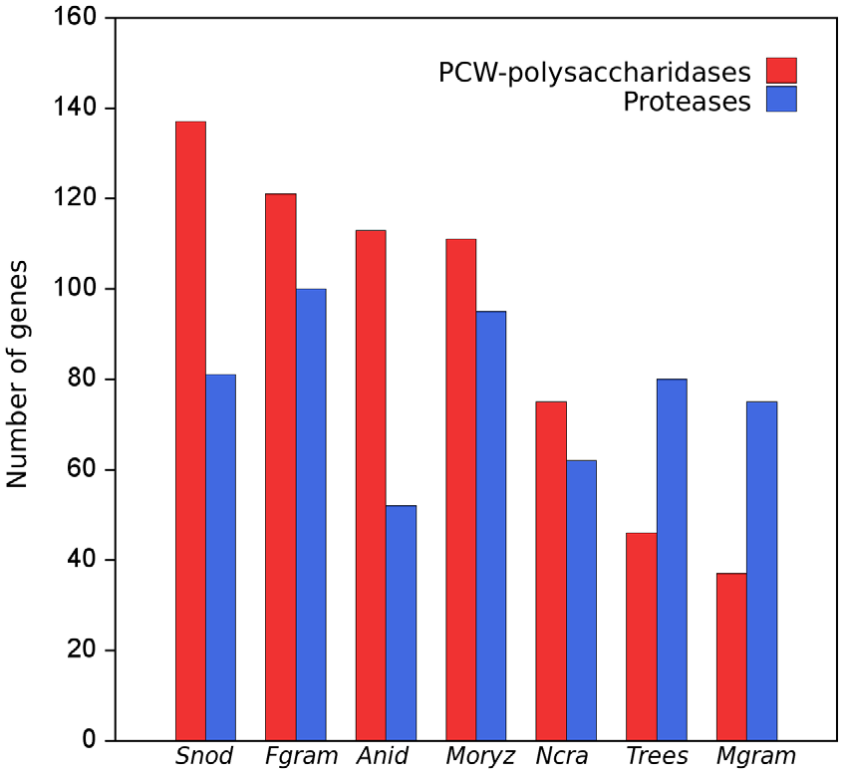
Numbers of genes for proteases and plant cell wall (PCW) degrading polysaccharidases in the genomes of seven fungi with sequenced genomes. Genes for PCW-polysaccharidases were severely reduced in the genome of *Mycosphaerella graminicola* but proteases were about the same. The overall profile of the enzymes in *M. graminicola* was most similar to that of *T. reesei* than to any of the other plant pathogens. Species analyzed included the saprophytes *Aspergillus nidulans* (Anid), *Neurospora crassa* (Ncra), and *Trichoderma reesei* (Trees), and the plant pathogens *Fusarium graminearum* (Fgram), *Mycosphaerella graminicola* (Mgram), *Magnaporthe oryzae* (Moryz), and *Stagonospora nodorum* (Snod).

Generally, gene families involved in cell wall degradation are expanded in fungal plant pathogens [24]–[25]. However, in *M. graminicola*, gene families characterized by the Carbohydrate-Active Enzyme database (CAZy) [26] as plant cell wall polysaccharidases were severely reduced in size (Figure 6). According to the CAZy analysis, the genome of *M. graminicola* contains fewer genes for cellulose degradation than those of six other fungi with sequenced genomes including both grass pathogens and saprophytes (Table 3), and only about one-third as many genes for cell wall degradation in total compared to the other plant pathogens (Table S4). This reduction in CAZymes in *M. graminicola* was very visible when the putative genes were divided based on polysaccharide substrate (Table S4). In addition, genes involved in appressorium formation, which are required for pathogenesis of many plant pathogens including *Magnaporthe oryzae* [27], were absent or reduced in the *Mycosphaerella graminicola* genome, reflecting its alternative host-penetration strategy.

**Table 3:** Numbers of predicted enzymes degrading cellulose across seven ascomycete species with sequenced genomes.

	Saprophytes^a^			Pathogens^a^			
CAZy family^b^	Anid	Ncra	Trees	Fgram	Mgram	Moryz	Snod
GH5 cellulases^c^	3	1	2	2	0	2	3
GH6	2	3	1	1	0	3	4
GH7	3	5	2	2	1	6	5
GH12	1	1	2	4	1	3	4
GH45	1	1	1	1	1	1	3
GH61	9	14	3	15	2	17	30
GH74	2	1	1	1	0	1	0
CBM1	8	19	15	12	0	22	13
Total cellulases	29	45	27	38	5	55	62

a Species analyzed included the saprophytes *Aspergillus nidulans* (Anid), *Neurospora crassa* (Ncra), and *Trichoderma reesii* (Trees), and the plant pathogens *Fusarium graminearum* (Fgram), *Mycosphaerella graminicola* (Mgram), *Magnaporthe oryzae* (Moryz), and *Stagonospora nodorum* (Snod).

b Families defined in the Carbohydrate-active enzymes database (www.cazy.org).

c GH5 is a family containing many different enzyme activities; only those targeting cellulose are included.

To further analyze the mechanism of stealth pathogenesis, we profiled the growth on polysaccharides of *M. graminicola* compared to *Stagonospora nodorum* and *Magnaporthe oryzae*, two pathogens of the cereals wheat and rice, respectively, with sequenced genomes (Figure S12). Growth of *M. graminicola* corresponded with the CAZy annotation for a strongly reduced number of genes encoding putative xylan-degrading enzymes. Furthermore, the CAZy annotation demonstrated that *M. graminicola* contains a much smaller set of glycoside hydrolases, carbohydrate esterases, and carbohydrate binding modules (CBMs) compared to the other two cereal pathogens (Table S5). The strong reduction of CBMs in *M. graminicola* suggests a different strategy in the degradation of plant cell walls compared to the other two species. The *M. graminicola* genome is particularly depauperate for enzymes degrading cellulose, xylan and xyloglucan compared to the other two species, so is very atypical for a cereal pathogen.

A possible mechanism of stealth pathogenesis was indicated by gene families that were expanded in the genome of *M. graminicola*. In comparative analyses of gene families and PFAM domains with several other fungi, the most striking expansions were observed for peptidases (M3, S28, pro-kuma, M24, metalloendopeptidase, metalloproteinase) and alpha amylases (glycoside hydrolase family 13) (Tables S6 and S7). This suggests that alternative nutrition sources during the biotrophic phase of infection may be proteins which are available in the apoplast, or possibly starch from chloroplasts that are released early in the infection process [5]. Overall, these analyses revealed that the genome of *M. graminicola* differs significantly from those of other cereal pathogens with respect to genes involved in plant penetration as well as polysaccharide and protein degradation (Figure 6, Table 3), which most likely reflects its stealthy mode of pathogenesis.

Differences in gene expression during the different stages of infection were evident from an analysis of EST sequences [9] from wheat leaves 5, 10 and 16 days after inoculation (DAI) with *M. graminicola*. Most genes were present at only one sampling time with little overlap, particularly between the library from the biotrophic stage of infection (5 DAI) compared to the other two (Figure S13A). Lack of overlap extended to a library from minimal medium minus nitrogen to simulate the nitrogen starvation thought to occur during infection (Figure S13B). Expression of genes for cell wall-degrading enzymes also was reduced during the biotrophic stage of infection [9], consistent with the stealth-pathogenicity hypothesis.

## Discussion

The dispensome as defined here includes all parts of the genome that can be missing in field or progeny isolates with no obvious effects on fitness in axenic culture, on a susceptible host or during mating. For *M. graminicola*, this includes the eight known dispensable chromosomes in isolate IPO323 plus any others that may be discovered in the future. The core genome consists of all chromosomes that are always present in field and progeny isolates, presumably because they contain genes that are vital for survival so cannot be lost. Both core and dispensable chromosomes may be present in two or possibly more copies, but core chromosomes are never absent.

The dispensome of *M. graminicola* is very different from the supernumerary or B chromosomes in plants and some animals. The B chromosomes of plants contain few if any genes and are composed mostly of repetitive elements assembled from the A chromosomes. They may have a negative effect on fitness [28] and appear to be maintained primarily by meiotic drive [29]. In contrast, the dispensome of *M. graminicola* contains many unique and redundant genes and is not maintained by meiotic drive, as individual chromosomes are lost readily during meiosis [11].

Dispensable chromosomes have been reported in other fungi but they are significantly fewer and larger (from 0.7 to 4.9 Mb with an average of about 1.5 to 2.0 Mb) than those in *M. graminicola* (from 0.42 to 0.77 Mb) and mostly are composed of repetitive DNA with few known genes [30]. Unlike the dispensome of *M. graminicola*, the few genes on dispensable chromosomes in other fungi often are pathogenicity factors [31]–[33] and whole chromosomes may be transferred asexually [34]. Dispensable chromosomes in other fungi are different from the dispensome of *M. graminicola* except for the conditionally dispensable or lineage-specific chromosomes reported recently in *Nectria haematococca* (asexual stage: *Fusarium solani*) and other species of Fusarium [35]–[36], which also were different from core chromosomes in structure and gene content and contained numerous unique genes. However, unlike those in *M. graminicola*, dispensable chromosomes of Fusarium species had clear functions in ecological adaptation, were transferred more or less intact among closely related species [35] and did not show extensive recombination with core chromosomes.

The high instability of the *M. graminicola* dispensome during meiosis and mitosis would cause it to be eliminated unless it provided a selective advantage to the pathogen at least under some conditions. The unique genes with annotations indicated possible functions in transcription or signal transduction. There also was an enrichment for predicted pre-milRNAs, which may indicate that parts of the dispensome are involved in gene regulation. Based on dispensable chromosomes in other plant pathogens, genes on the dispensome were expected to be involved with host adaptation or pathogenicity, yet so far no genes for pathogenicity or fitness of *M. graminicola* have been mapped to the dispensome [37]. A more interesting possibility is that the dispensome facilitates high recombination among chromosomes and could provide a repository of genes that may be advantageous under certain environmental conditions. This hypothesis should be tested by additional experimentation.

A recent comparison of the *M. graminicola* genome with that of its closest known relative, the unnamed species S1 from wild grasses in Iran, identified probable homologs for all of the dispensome chromosomes in the sibling species except for chromosome 18 [38]. These putative homologs presumably are dispensable also in species S1, but this has not been proven and only one isolate has been sequenced. Species S1 and *M. graminicola* are thought to have diverged approximately 10,500 years ago [39], concomitant with the domestication of wheat as a crop. Therefore, unlike dispensable chromosomes in other fungi, the dispensome of *M. graminicola* appears to be relatively ancient and has survived at least one speciation event. Analyses of two recently sequenced Dothideomycetes with Mycosphaerella sexual stages, *M. pini* (asexual stage: *Dothistroma septosporum*) and *M. populorum* (asexual stage: *Septoria musiva*), showed that they contained clear homologs of all of the core chromosomes of *M. graminicola*, but none of their chromosomes corresponded to the dispensome (B. Dhillon and S. B. Goodwin, unpublished). Taken together, these observations indicate that the dispensome of *M. graminicola* most likely was acquired prior to its divergence from a common ancestor with species S1 more than 10,000 years ago, but after the split of the *M.graminicola*-S1 lineage from that which gave rise to *M. pini* and *M. populorum*. The mechanism for the longevity of this dispensome with no obvious effects on fitness is not known.

More than half of the genes on the dispensome and almost all of the transposons also were present on core chromosomes. Moreover, there was no increase in gene numbers so a simple transfer of chromosomes from another species does not explain all of the observations. Instead, we propose a new model for the origin of dispensable chromosomes in *M. graminicola* by horizontal transfer followed by degeneration and extensive recombination with core chromosomes. The tight clustering of the dispensable chromosomes in the PCAs, with the possible exception of chromosome 14, indicates that they probably came from the same donor species. However, it is difficult to explain why they are so numerous. The most likely mechanism of horizontal transfer is via a sexual or somatic fusion with another species that had eight or more chromosomes, in which only a few genes were maintained on each donated chromosome. Chromosome segments that were redundant with the core set could be eliminated, leaving only those that are unique or that could confer some sort of selective advantage to the individual or to the dispensome. The fitness advantage could be transitory or occur only under certain conditions to allow those chromosomes to be dispensable, at least on an individual or population basis. Another possibility is that the numerous dispensable chromosomes are fragments from one or two larger chromosomes that were broken, acquired additional telomeres and lost content to result in their current, reduced complements of genes. High recombination within chromosomes and transfer of content between the donor and host chromosomes must have occurred to explain the observed pattern of shared genes.

The recombination hypothesis is supported by degenerated copies of some unique genes that were found on core chromosomes. These most likely represent genes that were copied from core to dispensable chromosomes, after which the copy on the core chromosome became inactivated, probably by RIP. Duplication, diversification and differential gene loss were proposed recently as the origin of lineage-specific gene islands in *Aspergillus fumigatus* [40], but that process seems to be very different from what occurred in *M. graminicola*. In *A. fumigatus*, large blocks of genes with synteny to other chromosomes were found, the opposite of what was seen for *M. graminicola*. The origin and evolution of the dispensome in *M. graminicola* seems to be very different from those reported for dispensable chromosomes in other fungi [35]. Unlike other fungi in which single chromosomes seem to have been transferred recently, the dispensome of *M. graminicola* most likely originated by ancient horizontal transfer of many chromosomes thousands of years ago. So far it is not known to be conditionally dispensable, unlike dispensable chromosomes in other fungi, which have clear roles in ecological adaptation.

The mesosyntenic analyses provided a new approach that complements the use of genetic linkage maps to support whole-genome assembly. Gene content was highly conserved on syntenic chromosomes in the two distantly related species, but there was little or no conservation of gene order or orientation. The comparison of the version 1 assembly of *M. graminicola* with the related *S. nodorum* genome sequence indicated scaffolds that should be merged and others that were erroneously assembled into one scaffold. Hence, mesosynteny validated the high-density genetic analyses and may provide an additional tool for whole-genome assembly for fungi where linkage maps do not exist or cannot be generated. Groups of genes in *S. nodorum* that corresponded to more than one group in *M. graminicola* may indicate scaffolds that should be joined in *S. nodorum* or, more likely, may reflect chromosomal rearrangements that have occurred since the divergence of *S. nodorum* and *M. graminicola* from an ancient common ancestor.

Considering their early divergence [41] relative to species within the same genus, the degree of mesosyntenic conservation between *M. graminicola* and *S. nodorum* is striking. However, it is very surprising that the synteny only applied to gene content but not order or orientation. In comparisons between other organisms, synteny plots usually yield diagonal lines even between unrelated species such as humans and cats [42]. The lack of diagonal lines in the comparisons of *S. nodorum* with *M. graminicola* indicate a high rate of shuffling of genes on chromosomal blocks that have remained constant over long periods of evolutionary time. The mechanism by which these small chromosomal rearrangements occur is not known.

The greatly reduced number of cell wall-degrading enzymes (CWDEs) in the genome of *M. graminicola* compared with other sequenced fungal genomes might be an evolutionary adaptation to avoid detection by the host during its extended, biotrophic latent phase and thus evade plant defenses long enough to cause disease. Similar loss of CWDEs in the ectomycorrhizal fungus *Laccaria bicolor* was thought to represent an adaptation to a symbiotic lifestyle [43]. Based on these results we propose a novel, biphasic mechanism of stealth pathogenesis. During penetration and early colonization, *M. graminicola* produces a reduced set of proteins that facilitate pathogenicity and function as effectors in other fungi. Instead of the usual carbohydrate metabolism, nutrition during the extended biotrophic phase may be by degradation of proteins rather than carbohydrates in the apoplastic fluid and intercellular spaces. The large number of proteases expressed during the early stages of the infection process supports this hypothesis. The biotrophic phase terminates by a switch to necrotrophic growth, production of specific cell wall-degrading enzymes and possibly by triggering programmed cell death [5], [9]–[10].

Stealth biotrophy raises the intriguing possibility that *M. graminicola* and possibly other Dothideomycetes may have evolved originally as endophytes or could be evolving towards an endophytic lifestyle. The finished genome of *M. graminicola* provides a gold standard [12] for this class of fungi, which is the largest and most ecologically diverse group of Ascomycetes with approximately 20,000 species, classified in 11 orders and 90 families, and provides a huge advantage for comparative genomics to identify the genetic basis of highly divergent lifestyles.

## Materials and Methods

### Biological material

*Mycosphaerella graminicola* isolates IPO323 and IPO94269 are Dutch field strains that were isolated in 1984 and 1994 from the wheat cultivar Arminda and an unknown cultivar, respectively. Isolate IPO95052 was isolated from a durum (tetraploid) wheat sample from Algeria. All isolates are maintained at the CBS-KNAW Fungal Biodiversity Centre of the Royal Netherlands Academy of Arts and Sciences (Utrecht, the Netherlands) under accession numbers CBS 115943 (IPO323), CBS 115941 (IPO94269) and CBS 115942 (IPO95052). Mycelia of each isolate were used to inoculate 200 mL of YG broth (10 g of yeast extract and 30 g of glucose per L) and were cultured until cloudy by shaking at 120 rpm at 18°C, after which the spores were lyophilized, 50 mg of lyophilised spores were placed in a 2-mL tube and ground with a Hybaid Ribolyser (model n° FP120HY-230) for 10 s at 2500 rpm with a tungsten carbide bead. DNA was extracted using the Promega Wizard Magnetic DNA Purification system for food according to instructions provided by the manufacturer.

### Initial sequencing and assembly

Whole-genome shotgun (WGS) sequencing of the genome of *M. graminicola* used three libraries with insert sizes of 2–3, 6–8, and 35–40 kb. The sequenced reads were screened for vector using cross_match, trimmed for vector and quality, and filtered to remove reads shorter than 100 bases. WGS assembly was done using Jazz, a tool developed at the JGI [44]. After excluding redundant and short scaffolds, the assembly v1.0 contained 41.2 Mb of sequence in 129 scaffolds, of which 4.0 Mb (7.5%) was in gaps (Table S8). The sequence depth derived from the assembly was 8.88±0.04.

### Gap closure and finishing

To perform finishing, the *M. graminicola* WGS assembly was broken down into scaffold-size pieces and each piece was reassembled with phrap. These scaffold pieces were then finished using a Phred/Phrap/Consed pipeline. Initially, all low-quality regions and gaps were targeted with computationally selected sequencing reactions completed with 4∶1 BigDye terminator: dGTP chemistry (Applied Biosystems). These automated rounds included resequencing plasmid subclones and walking on plasmid subclones or fosmids using custom primers.

Following completion of the automated rounds, a trained finisher manually inspected each assembly. Further reactions were then manually selected to complete the genome. These included additional resequencing reactions and custom primer walks on plasmid subclones or fosmids as described above guided by a genetic map of more than 2,031 sequenced markers plus paired-end reads from a library of Bacterial Artificial Chromosome clones. Smaller repeats in the sequence were resolved by transposon-hopping 8-kb plasmid clones. Fosmid and BAC clones were shotgun sequenced and finished to fill large gaps, resolve larger repeats and to extend into the telomere regions.

Each assembly was then validated by an independent quality assessment. This included a visual examination of subclone paired ends using Orchid (http://www-hagsc.org), and visual inspection of high-quality discrepancies and all remaining low-quality areas. All available EST resources were also placed on the assembly to ensure completeness. The finished genome consists of 39,686,251 base pairs of finished sequence with an estimated error rate of less than 1 in 100,000 base pairs. Genome contiguity is very high with a total of 21 chromosomes represented, 19 of which are complete and 20 of which are sequenced from telomere to telomere.

### Genome annotation

Both draft (v1.0) and finished (v2.0) assemblies of *M. graminicola* were processed using the JGI annotation pipeline, which combines several gene predictors:1) putative full-length genes from EST cluster consensus sequences; 2) homology-based gene models were predicted using FGENESH+ [45] and Genewise [46] seeded by Blastx alignments against sequences from the NCBI non-redundant protein set; 3) *ab initio* gene predictor FGENESH [45] was trained on the set of putative full-length genes and reliable homology-based models. Genewise models were completed using scaffold data to find start and stop codons. ESTs were used to extend, verify, and complete the predicted gene models. Because multiple gene models per locus were often generated, a single representative gene model for each locus was chosen based on homology and EST support and used for further analysis. Those comprised a filtered set of gene models supported by different lines of evidence. These were further curated manually during community annotation and used for analysis.

All predicted gene models were annotated using InterProScan [47] and hardware-accelerated double-affine Smith-Waterman alignments (www.timelogic.com) against the SwissProt (www.expasy.org/sprot) and other specialized databases such as KEGG [48]. Finally, KEGG hits were used to map EC numbers (http://www.expasy.org/enzyme/), and Interpro hits were used to map GO terms [49]. Predicted proteins also were annotated according to KOG [50], [51] classification.

Following the machine annotation, manual validation and correction of selected gene sets was performed by more than 30 annotators through a jamboree held at the JGI facilities in Walnut Creek, California, USA. Annotators were trained by JGI staff and continue to make modifications as necessary.

Potential microRNA-like small RNA (milRNAs) loci were annotated using the INFERNAL software tool and based on 454 microRNA families (covarion models) from the RFAM database version 9.1 [52]. milRNAs were predicted if their scores were higher than thresholds, defined individually for each family, in the same way as PFAM domains are predicted.

Experimental validation of the predicted milRNAs was done by sequencing of an RNA library Total RNA was isolated from spores germinated on water agar of *M. graminicola* isolate IPO323. A small RNA library was prepared according to the protocol for Illumina sequencing; small RNAs from 16–∼50 nt were isolated from gels, sequenced with an Illumina/Solexa single read DNA 50 cycles Genome Analyzer II, and compared by BLAST search against the list of 535 predicted pre-milRNAs from the genome sequence.

Assembly and annotations of the *M. graminicola* finished genome are available from the JGI Genome Portal at http://www.jgi.doe.gov/Mgraminicola and were deposited at DDBJ/EMBL/GenBank under the project accession ACPE00000000.

### Microarray analyses

Whole-genome tiling microarrays were designed by choosing one 50-mer primer every 100 bases spanning the entire finished genome. The arrays were manufactured and hybridized by the Nimblegen Corporation with total DNA extracted from each field isolate.

### Principal component analyses of core and dispensable chromosomes

The CodonW package (http://codonw.sourceforge.net/) was used for correspondence analysis of codon usage, which mathematically is identical to principal component analysis. CodonW requires as an input a set of coding sequences, usually of individual genes. For chromosome-level analyses coding sequences from the frozen gene catalog models for each chromosomes were concatenated, forming 21 ‘superORFs’, one for each chromosome. Because partial models may introduce some potential frameshifts with internal stop codons they were removed from the analysis; this did not affect the results as their total number is low. CodonW has no graphical outputs, so they were used as inputs for scatter plots in R (http://www.r-project.org/).

For *M. graminicola* only a similar analysis was done for repeats. RepeatScout was run on the genome to produce a set of *ab initio*-identified repeat sequences. From that set 81 distinct repeat sequences, each with an occurrence exceeding 20 times in the genome, were extracted. For each chromosome a vector of length 81 was calculated with the relative frequency of each repeat. A PC analysis was run on the resulting vectors using the standard principal component function pcomp in R. Separation at the repeat level means that these chromosomes have distinct evolutionary profiles not only on the protein-coding level, but also on other parts of the chromosomes, suggesting that entire chromosomes may be transferred horizontally.

### Mesosynteny

Dot plots were generated via MUMMER 3.0 [53] with data derived from default PROmer comparisons between the *M. graminicola* genome assembly versions 1 and 2 (http://genome.jgi-psf.org/Mycgr3/Mycgr3.home.html) and *S. nodorum* SN15 assembly version 2 [54], available under GenBank accessions CH445325–CH445384, CH445386–CH445394 and CH959328–CH959365, or AAGI00000000. Additional comparisons and statistical analyses were made with custom-designed perl scripts.

Data from the *M. graminicola* version 2 comparison with *S. nodorum* were used to test the efficacy of mesosyntenic comparisons to assist the completion of fungal genomes. The mesosynteny-based prediction of scaffold joining involved 3 stages: determining the percent coverage of scaffolds/chromosomes for each scaffold/chromosome pair (i.e., a function of the number of ‘dots’ per ‘block’); determining which scaffold/chromosome pairs were significantly related and forming groups of joined scaffolds; and filtering out background levels of similarity due to sequence redundancy and incomplete genome assemblies.

Coordinates of homologous regions were obtained from PROmer coordinate outputs (MUMMER 3.0) and used to determine the percent of sequence covered by matches to a sequence from the alternate genome for each sequence pair. Where match coordinates overlapped on the sequence of interest, those matches were merged into a single feature to avoid redundancy. A perl script for conversion of PROmer coordinate outputs to a table of percent coverage is available on request.

Coverage values for each *M. graminicola*-*S. nodorum* sequence pair were subject to a binomial test for significance. The threshold for significance (*Psig*)≥0.95 was:
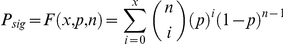
where *x* is the percent coverage, *n* equals 100, and *p* is the probability of chromosome homology.

The probability of chromosome homology (*p*) was equal to 1/(21×19), which was derived from the number of *M. graminicola* chromosomes (21) and the approximate PFGE estimate of *S. nodorum* chromosomes (19) [55]. This is the likelihood that a given sequence pair represents related chromosomes. This model assumes that no whole-genome/chromosome duplication events have occurred previously between either fungal genome since divergence from their last common ancestor.

The significance of percent coverage (*Psig*) was tested bidirectionally for each sequence pair (i.e., for sequence pair A–B, both coverage of sequence A by B and coverage of sequence B by A were tested). Sequence pairs were significantly related if a test in either direction was successful. A minimum length threshold of 1 kb was also imposed for both sequences. Where multiple scaffolds of *M. graminicola* were significantly related to the same *S. nodorum* scaffold, those *M. graminicola* scaffolds formed a ‘joined group’ of candidates for representation of the same chromosome.

All possible paired combinations of *M. graminicola* scaffolds present within predicted joined groups were subject to filtering for high levels of background similarity as follows:
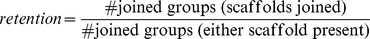
The retention score is a measure of the reliability of scaffold join relationships. Joins between *M. graminicola* scaffold pairs with retention scores <0.25 were discarded.

### CAZy annotation and growth profiling

Annotation of carbohydrate-related enzymes was performed using the Carbohydrate-Active Enzyme database (CAZy) annotation pipeline [26]. BLAST was used to compare the predicted proteins of *M. graminicola* to a collection of catalytic and carbohydrate-binding modules derived from CAZy. Significant hits were compared individually by BLAST to assign them to one or more CAZy families. Ambiguous family attributions were processed manually along with all identified models that presented defects (deletions, insertions, splicing issues, etc.).

Growth profiling of *S. nodorum* and *M. graminicola* was on *Aspergillus niger* minimal medium [56]. Cultures were grown at 25 degrees for seven days after which pictures were taken for growth comparison. Carbon sources used were: glucose (Sigma); soluble starch (Difco); alpha-cellulose (Sigma); Guar Gum (Sigma, galactomannan); Oat spelt xylan (Sigma); and Apple Pectin (Sigma).

### Genome structure analyses

Comparisons of sequence content between core and dispensable chromosomes was with Circos [57]. This tool draws ribbons connecting sequences that align in different data sets.

## Supporting Information

Dataset S1 The method and calculations for using mesosynteny to predict scaffold joins from version 1 to version 2 of the *Mycosphaerella graminicola* genomic sequence.
(XLS)

Figure S1 Aspects of the *in vitro* and *in vivo* lifestyle of *Mycosphaerella graminicola*. 1. Typical colony appearance of *M. graminicola* isolates grown under light (upper two rows) and dark (lower low) conditions. Light stimulates yeast-like growth whereas darkness induces filamentous growth. 2. Close-up of yeast-like growth on V8 agar. 3. *In vitro* production of asexual fructifications (pycnidia; arrow) on wheat leaf extract agar. 4. Penetration of a wheat leaf stoma (arrow) by a pycnidiospore germ tube. 5. Simultaneous penetration of a wheat leaf stoma by three germ tubes of sexual airborne ascospores (arrows) that are transported over vast distances. 6. Colonization of the mesophyll tissue by an intercellular hypha (arrows) during the symptomless biotrophic phase of pathogenesis. 7. Initiation (arrow head) of a pycnidium in the substomatal cavity of a wheat leaf. 8. Ripe pycnidia in a primary leaf of a susceptible wheat seedling. High humidity stimulates the extrusion of cyrrhi, tendril-like mucilages containing asexual pycnidiospores that are rain-splash dispersed over short distances. 9. Typical infection of the primary leaf of a resistant cultivar. Note the low fungal density in the apoplast (arrow) and the response of the mesophyll cells (arrow head), particularly the chloroplasts, to the presence of intercellular hyphae. 10. Typical symptoms on a primary seedling leaf of a highly susceptible wheat cultivar. 11. Typical response on a primary leaf of a highly resistant wheat cultivar. 12. Adult-plant evaluation plots are inoculated at the adult plant stage with individual isolates using air-driven equipment. 13. Symptoms on an adult plant flag leaf after field inoculations. 14. Symptoms on a naturally infected adult plant flag leaf.
(TIF)

Figure S2 The 21 chromosomes of the *Mycosphaerella graminicola* genome drawn to scale. Red indicates regions of single-copy sequence; repetitive sequences are in shown blue. Chromosome 1 is almost twice as long as any of the others. The core chromosomes 1–13 are the largest. Dispensable chromosomes 14–21 are smaller than the core chromosomes and have a higher proportion of repetitive DNA as indicated by the blue bands.
(TIF)

Figure S3 Features of chromosome 14, the largest dispensable chromosome of *Mycosphaerella graminicola*, and alignment to genetic linkage maps. A, Plot of GC content. Areas of low GC usually correspond to regions of repetitive DNA. B, Repetitive regions of the *M. graminicola* genome. C, Single-copy (red) regions of the *M. graminicola* genome. D, Locations of genes for proteins containing signal peptides. E, Locations of homologs of pathogenicity or virulence genes that have been experimentally verified in species pathogenic to plant, animal or human hosts. F, Approximate locations of quantitative trait loci (QTL) for pathogenicity to wheat. G, Alignments between the genomic sequence and two genetic linkage maps of crosses involving isolate IPO323. Top half, Genetic linkage map of the cross between IPO323 and the Algerian durum wheat isolate IPO95052. Bottom half, Genetic linkage map of the cross between bread wheat isolates IPO323 and IPO94269. The physical map represented by the genomic sequence is in the center. Lines connect mapped genetic markers in each linkage map to their corresponding locations on the physical map based on the sequences of the marker loci. Very few secreted proteins (track D) or pathogenicity-related genes (E) and no pathogenicity QTL mapped to the dispensome.
(TIF)

Figure S4 Principal Component Analysis of codon usage in 21 chromosomes of the *Mycosphaerella graminicola* finished genome. Factor 1 gave good discrimination between core (blue circles) and dispensable (red) chromosomes.
(TIF)

Figure S5 Examples of unique genes on dispensable chromosomes with an inactivated copy on a core chromosome. A unique gene on chromosome 14 and two on chromosome 18 showed excellent alignments to footprints of genes on chromosome 1. The copies on chromosome 1 matched those on the dispensable chromosomes with an expected value of 1×10^−5^ or better, but contained numerous stop codons indicating that they were pseudogenes and possibly could have been the progenitor copies for the intact, unique genes on dispensable chromosomes 14 and 18. The graphs above chromosome 14 and below chromosome 18 indicate GC content.
(TIF)

Figure S6 Examples of amino acid alignments between protein sequences of unique genes on dispensable chromosomes to their inactivated putative homologs on core chromosomes. A, A unique gene on dispensable chromosome 14 aligned to a footprint of its homologous pseudogene on core chromosome 1. B and C, Alignments between two genes on dispensable chromosome 18 to homologous pseudogenes on core chromosome 1. Identical amino acids are shaded blue. Stop codons in pseudogenes are indicated by X and are shaded red. Details are provided beneath each alignment. Each unique gene is at least 26% identical and 46% similar to its putative homolog.
(TIF)

Figure S7 Analysis of genes and repetitive DNAs that are shared between dispensable chromosome 14 and the 13 core chromosomes of *Mycosphaerella graminicola*. Each chromosome is drawn to scale as a numbered bar around the outer edge of the circle. Lines connect regions of 100 bp or larger that are similar between each core chromosome and the corresponding region on chromosome 14 at 1×e^−5^ or lower. Chromosome 14 contains parts of all of the core chromosomes that are mixed in together with no synteny. Genes on the other dispensable chromosomes were not included in this analysis.
(TIF)

Figure S8 Analysis of genes that are shared between each of the nine largest core chromosomes (1–9) and all other chromosomes of the *Mycosphaerella graminicola* genome. Each chromosome is drawn to scale as a numbered bar around the outer edge of the circle. Lines connect regions of 100 bp or larger that are similar between the indicated core chromosome and each of the remaining 20 chromosomes at 1×e^−5^ or lower. Each chromosome contains parts of all of the other chromosomes mixed in together with no synteny. Genes on the 12 smallest chromosomes were similar but are not shown.
(TIF)

Figure S9 Principal Component Analysis of codon usage. A, in 21 chromosomes of the *Mycosphaerella graminicola* finished genome after simulated RIPping. B, in 21 chromosomes of the *M. graminicola* finished genome after simulated deRIPping. C, of about 150 genes with shared putative homologs between the core and dispensable chromosomes of *M. graminicola*. D, of amino acid composition of about 150 genes with shared putative homologs between the core and dispensable chromosomes of *M. graminicola*. E, of all genes with shared putative homologs between the core and dispensable chromosomes of *M. graminicola*. F, of all genes on dispensable chromosomes with shared putative homologs on core chromosomes against all genes on the core chromosomes of *M. graminicola*.
(TIF)

Figure S10 Principal Component Analysis of codon usage. A,between the genomes of *M. graminicola* and *Stagonospora nodorum*. B, between the genomes of *M. graminicola* and *Aspergillus fumigatus*. Values for the chromosomes of *M. graminicola* are indicated by red circles, those for *S. nodorum* and *A. fumigatus* by green triangles.
(TIF)

Figure S11 Principal Component Analysis of repeats in 21 chromosomes of the *Mycosphaerella graminicola* finished genome. Core chromosomes (black circles) were clearly separated from the dispensome (red).
(TIF)

Figure S12 Growth of *Mycosphaerella graminicola*, *Stagonospora nodorum* and *Magnaporthe oryzae* (*M. grisea*) on glucose and several plant polysaccharides. Growth of *M. graminicola* was decreased on xylan, consistent with the CAZy annotation for fewer genes involved in degradation of that substrate.
(TIF)

Figure S13 Venn diagrams showing the expression of *Mycosphaerella graminicola* genes at different times during the infection process and with a sample grown *in vitro*. A, Libraries MgEST_08, MgEST_09, and MgEST_10 contain EST sequences from wheat leaf tissue collected at 5, 10 and 16 days after inoculation, respectively. B, four-way diagram with the same three in vitro-produced libraries plus *in vitro* library MgEST_05, grown on minimal medium minus nitrogen to mimic the early stages of the infection process.
(TIF)

Table S1 List of functional domains or other annotations for 65 genes on dispensable chromosomes 14–21 of the genome of *Mycosphaerella graminicola*.
(DOCX)

Table S2 Analysis of small RNA sequences (generated on the Illumina platform) for the presence of computationally predicted pre-microRNA-like (milRNA) sequences in germinated spores of *Mycosphaerella graminicola* isolate IPO323.
(DOCX)

Table S3 Best non-self BLAST hits for 654 called genes on dispensable chromosomes of *Mycosphaerella graminicola* queried with *tblastn* against a combined database containing the GenBank nt and EST datasets plus *M. graminicola* version 2.0 and *M. fijiensis* v1.0 from the Joint Genome Institute.
(DOCX)

Table S4 Numbers of predicted enzymes degrading hemicellulose, pectin and cutin across seven ascomycete species with sequenced genomes.
(DOCX)

Table S5 Total numbers of predicted CAZymes in *Mycosphaerella graminicola* and selected ascomycetes.
(DOCX)

Table S6 PFAM domains that are expanded in the genome of *Mycosphaerella graminicola* relative to those of five other Ascomycetes^a^ and two plant-pathogenic Stramenopiles^b^.
(DOCX)

Table S7 PFAM domains that are expanded in the genome of *Mycosphaerella graminicola* relative to those of five other Ascomycetes^a^ but not the two plant-pathogenic Stramenopiles^b^.
(DOCX)

Table S8 Assembly statistics for the *Mycosphaerella graminicola* version 1 (8.9× draft) and version 2 (finished) sequences compared to the 10× draft sequence of *Stagonospora nodorum*.
(DOCX)
