# Supplementary figures and images for: Finished Genome of the Fungal Wheat Pathogen Mycosphaerella graminicola Reveals Dispensome Structure, Chromosome Plasticity, and Stealth Pathogenesis
Source: PLoS Genet. 2011 Jun 9;7(6):e1002070. doi: 10.1371/journal.pgen.1002070 (PMC3111534; doi:10.1371/journal.pgen.1002070)

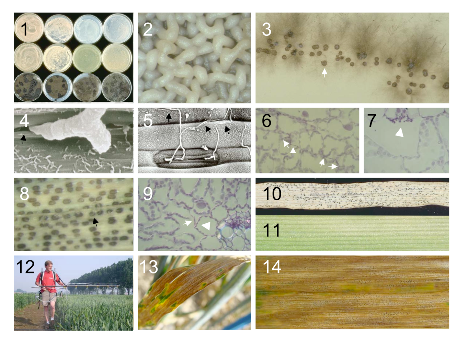

Supplement: Figure S1 — Aspects of the in vitro and in vivo lifestyle of Mycosphaerella graminicola. 1. Typical colony appearance of M. graminicola isolates grown under light (upper two rows) and dark (lower low) conditions. Light stimulates yeast-like growth whereas darkness induces filamentous growth. 2. Close-up of yeast-like growth on V8 agar. 3. In vitro production of asexual fructifications (pycnidia; arrow) on wheat leaf extract agar. 4. Penetration of a wheat leaf stoma (arrow) by a pycnidiospore germ tube. 5. Simultaneous penetration of a wheat leaf stoma by three germ tubes of sexual airborne ascospores (arrows) that are transported over vast distances. 6. Colonization of the mesophyll tissue by an intercellular hypha (arrows) during the symptomless biotrophic phase of pathogenesis. 7. Initiation (arrow head) of a pycnidium in the substomatal cavity of a wheat leaf. 8. Ripe pycnidia in a primary leaf of a susceptible wheat seedling. High humidity stimulates the extrusion of cyrrhi, tendril-like mucilages containing asexual pycnidiospores that are rain-splash dispersed over short distances. 9. Typical infection of the primary leaf of a resistant cultivar. Note the low fungal density in the apoplast (arrow) and the response of the mesophyll cells (arrow head), particularly the chloroplasts, to the presence of intercellular hyphae. 10. Typical symptoms on a primary seedling leaf of a highly susceptible wheat cultivar. 11. Typical response on a primary leaf of a highly resistant wheat cultivar. 12. Adult-plant evaluation plots are inoculated at the adult plant stage with individual isolates using air-driven equipment. 13. Symptoms on an adult plant flag leaf after field inoculations. 14. Symptoms on a naturally infected adult plant flag leaf. (TIF) [file pgen.1002070.s002.tif]

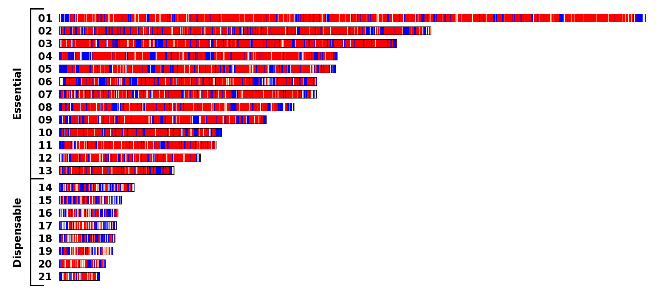

Supplement: Figure S2 — The 21 chromosomes of the Mycosphaerella graminicola genome drawn to scale. Red indicates regions of single-copy sequence; repetitive sequences are in shown blue. Chromosome 1 is almost twice as long as any of the others. The core chromosomes 1–13 are the largest. Dispensable chromosomes 14–21 are smaller than the core chromosomes and have a higher proportion of repetitive DNA as indicated by the blue bands. (TIF) [file pgen.1002070.s003.tif]

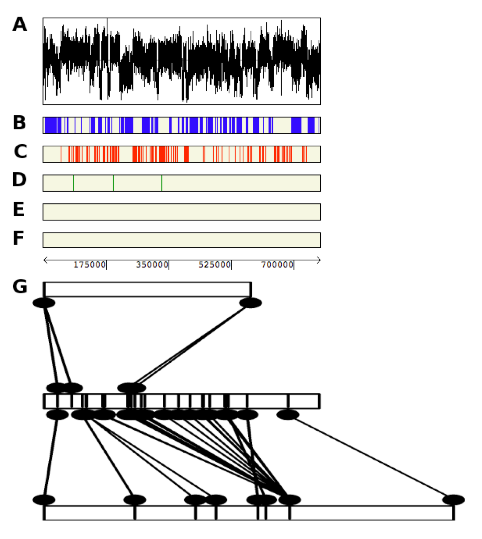

Supplement: Figure S3 — Features of chromosome 14, the largest dispensable chromosome of Mycosphaerella graminicola, and alignment to genetic linkage maps. A, Plot of GC content. Areas of low GC usually correspond to regions of repetitive DNA. B, Repetitive regions of the M. graminicola genome. C, Single-copy (red) regions of the M. graminicola genome. D, Locations of genes for proteins containing signal peptides. E, Locations of homologs of pathogenicity or virulence genes that have been experimentally verified in species pathogenic to plant, animal or human hosts. F, Approximate locations of quantitative trait loci (QTL) for pathogenicity to wheat. G, Alignments between the genomic sequence and two genetic linkage maps of crosses involving isolate IPO323. Top half, Genetic linkage map of the cross between IPO323 and the Algerian durum wheat isolate IPO95052. Bottom half, Genetic linkage map of the cross between bread wheat isolates IPO323 and IPO94269. The physical map represented by the genomic sequence is in the center. Lines connect mapped genetic markers in each linkage map to their corresponding locations on the physical map based on the sequences of the marker loci. Very few secreted proteins (track D) or pathogenicity-related genes (E) and no pathogenicity QTL mapped to the dispensome. (TIF) [file pgen.1002070.s004.tif]

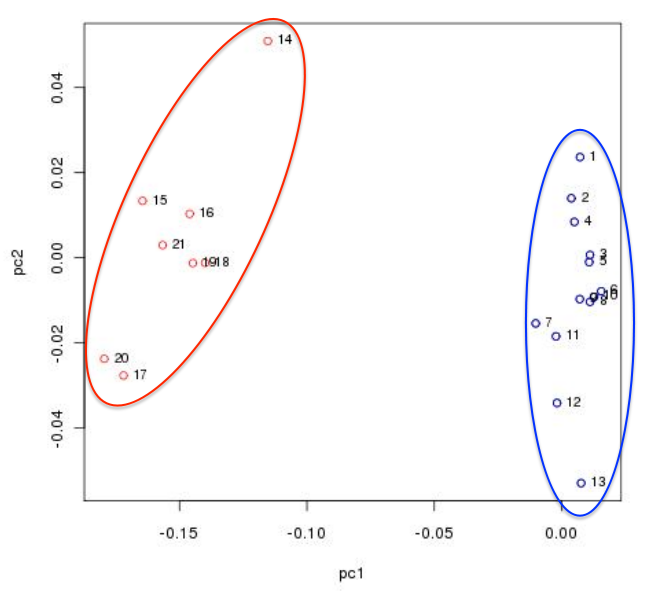

Supplement: Figure S4 — Principal Component Analysis of codon usage in 21 chromosomes of the Mycosphaerella graminicola finished genome. Factor 1 gave good discrimination between core (blue circles) and dispensable (red) chromosomes. (TIF) [file pgen.1002070.s005.tif]

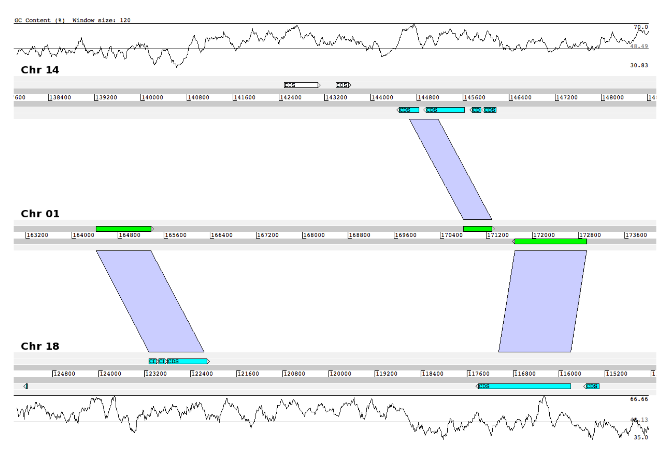

Supplement: Figure S5 — Examples of unique genes on dispensable chromosomes with an inactivated copy on a core chromosome. A unique gene on chromosome 14 and two on chromosome 18 showed excellent alignments to footprints of genes on chromosome 1. The copies on chromosome 1 matched those on the dispensable chromosomes with an expected value of 1×10^−5^ or better, but contained numerous stop codons indicating that they were pseudogenes and possibly could have been the progenitor copies for the intact, unique genes on dispensable chromosomes 14 and 18. The graphs above chromosome 14 and below chromosome 18 indicate GC content. (TIF) [file pgen.1002070.s006.tif]

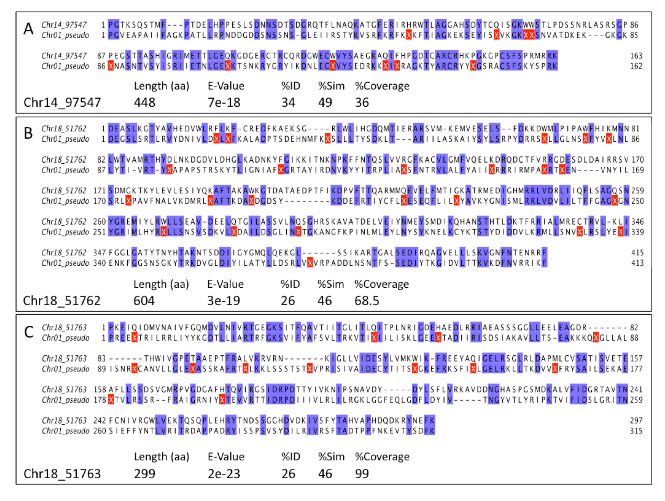

Supplement: Figure S6 — Examples of amino acid alignments between protein sequences of unique genes on dispensable chromosomes to their inactivated putative homologs on core chromosomes. A, A unique gene on dispensable chromosome 14 aligned to a footprint of its homologous pseudogene on core chromosome 1. B and C, Alignments between two genes on dispensable chromosome 18 to homologous pseudogenes on core chromosome 1. Identical amino acids are shaded blue. Stop codons in pseudogenes are indicated by X and are shaded red. Details are provided beneath each alignment. Each unique gene is at least 26% identical and 46% similar to its putative homolog. (TIF) [file pgen.1002070.s007.tif]

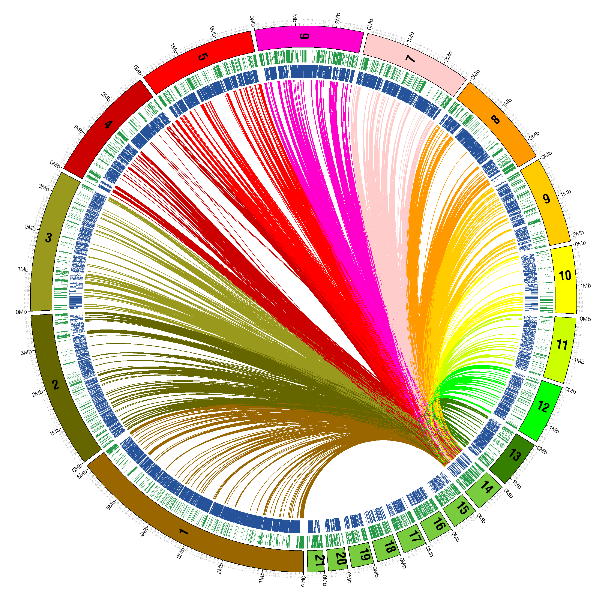

Supplement: Figure S7 — Analysis of genes and repetitive DNAs that are shared between dispensable chromosome 14 and the 13 core chromosomes of Mycosphaerella graminicola. Each chromosome is drawn to scale as a numbered bar around the outer edge of the circle. Lines connect regions of 100 bp or larger that are similar between each core chromosome and the corresponding region on chromosome 14 at 1×e−5 or lower. Chromosome 14 contains parts of all of the core chromosomes that are mixed in together with no synteny. Genes on the other dispensable chromosomes were not included in this analysis. (TIF) [file pgen.1002070.s008.tif]

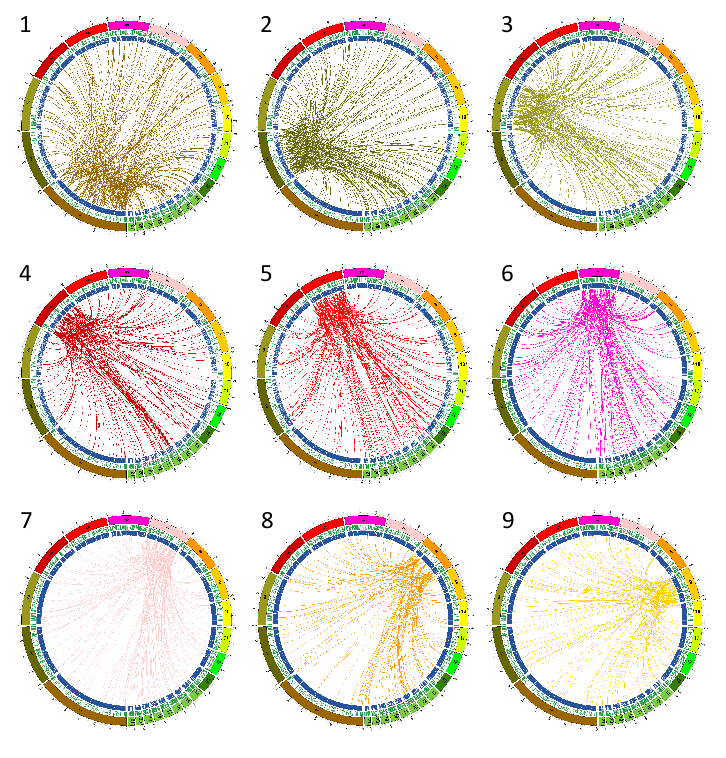

Supplement: Figure S8 — Analysis of genes that are shared between each of the nine largest core chromosomes (1–9) and all other chromosomes of the Mycosphaerella graminicola genome. Each chromosome is drawn to scale as a numbered bar around the outer edge of the circle. Lines connect regions of 100 bp or larger that are similar between the indicated core chromosome and each of the remaining 20 chromosomes at 1×e−5 or lower. Each chromosome contains parts of all of the other chromosomes mixed in together with no synteny. Genes on the 12 smallest chromosomes were similar but are not shown. (TIF) [file pgen.1002070.s009.tif]

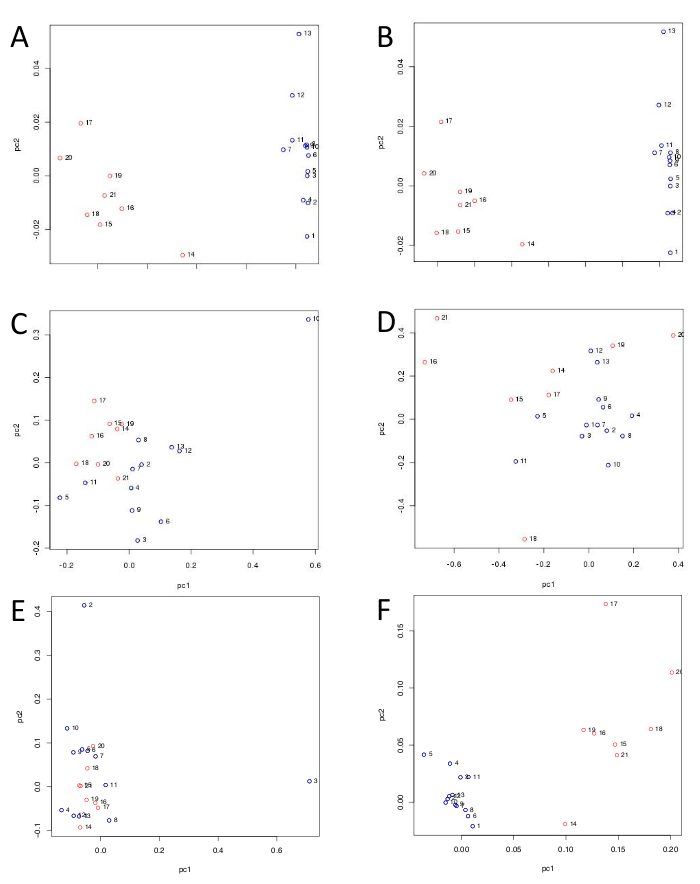

Supplement: Figure S9 — Principal Component Analysis of codon usage. A, in 21 chromosomes of the Mycosphaerella graminicola finished genome after simulated RIPping. B, in 21 chromosomes of the M. graminicola finished genome after simulated deRIPping. C, of about 150 genes with shared putative homologs between the core and dispensable chromosomes of M. graminicola. D, of amino acid composition of about 150 genes with shared putative homologs between the core and dispensable chromosomes of M. graminicola. E, of all genes with shared putative homologs between the core and dispensable chromosomes of M. graminicola. F, of all genes on dispensable chromosomes with shared putative homologs on core chromosomes against all genes on the core chromosomes of M. graminicola. (TIF) [file pgen.1002070.s010.tif]

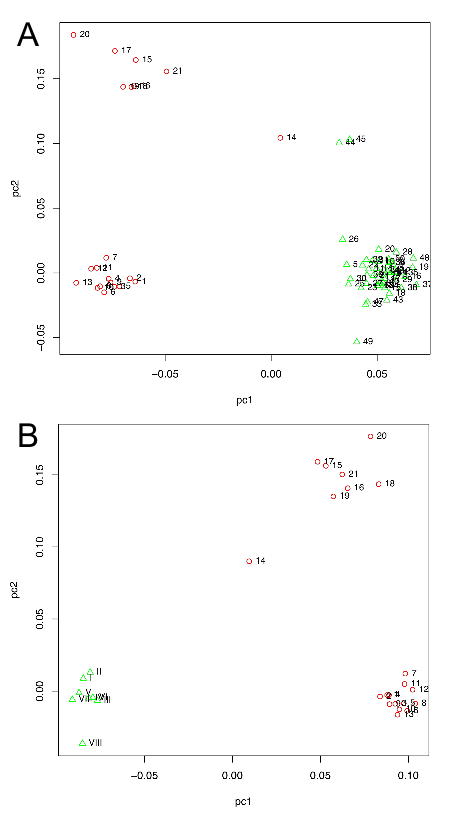

Supplement: Figure S10 — Principal Component Analysis of codon usage. A,between the genomes of M. graminicola and Stagonospora nodorum. B, between the genomes of M. graminicola and Aspergillus fumigatus. Values for the chromosomes of M. graminicola are indicated by red circles, those for S. nodorum and A. fumigatus by green triangles. (TIF) [file pgen.1002070.s011.tif]

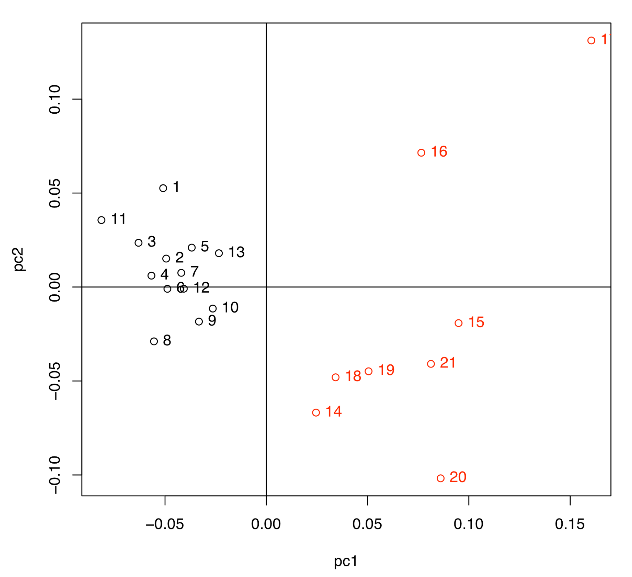

Supplement: Figure S11 — Principal Component Analysis of repeats in 21 chromosomes of the Mycosphaerella graminicola finished genome. Core chromosomes (black circles) were clearly separated from the dispensome (red). (TIF) [file pgen.1002070.s012.tif]

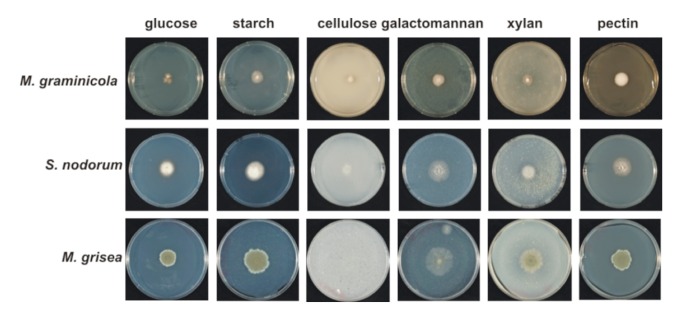

Supplement: Figure S12 — Growth of Mycosphaerella graminicola, Stagonospora nodorum and Magnaporthe oryzae (M. grisea) on glucose and several plant polysaccharides. Growth of M. graminicola was decreased on xylan, consistent with the CAZy annotation for fewer genes involved in degradation of that substrate. (TIF) [file pgen.1002070.s013.tif]

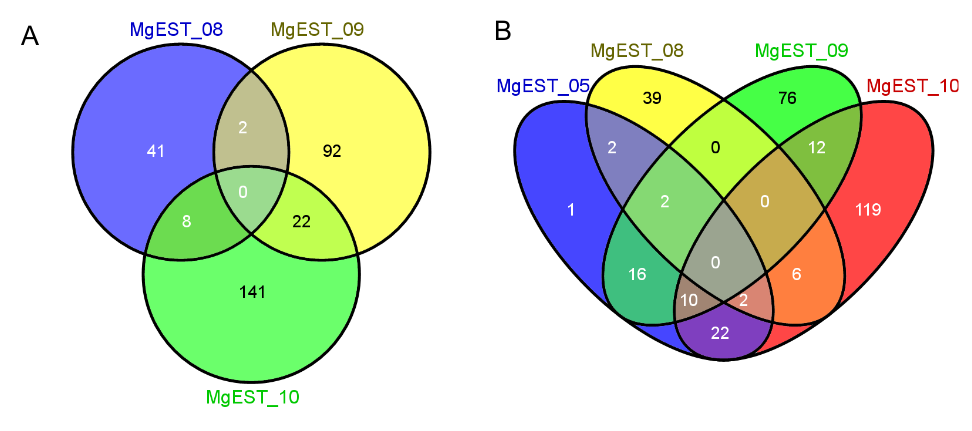

Supplement: Figure S13 — Venn diagrams showing the expression of Mycosphaerella graminicola genes at different times during the infection process and with a sample grown in vitro. A, Libraries MgEST_08, MgEST_09, and MgEST_10 contain EST sequences from wheat leaf tissue collected at 5, 10 and 16 days after inoculation, respectively. B, four-way diagram with the same three in vitro-produced libraries plus in vitro library MgEST_05, grown on minimal medium minus nitrogen to mimic the early stages of the infection process. (TIF) [file pgen.1002070.s014.tif]
