# Supplementary material for: Finished Genome of the Fungal Wheat Pathogen Mycosphaerella graminicola Reveals Dispensome Structure, Chromosome Plasticity, and Stealth Pathogenesis
Source: PLoS Genet. 2011 Jun 9;7(6):e1002070. doi: 10.1371/journal.pgen.1002070 (PMC3111534; doi:10.1371/journal.pgen.1002070)
Supplement: Table S1 — List of functional domains or other annotations for 65 genes on dispensable chromosomes 14–21 of the genome of Mycosphaerella graminicola. (DOCX) [file pgen.1002070.s015.docx]

**Table S1.** List of functional domains or other annotations for 65 genes on dispensable chromosomes 14-21 of the genome of *Mycosphaerella graminicola.*

| Location | Protein ID number^a^ | Annotation/domain information |
| --- | --- | --- |
| Chromosome 14 | 30708 | Protein kinase |
|  | 51580 | Kinesin, motor region |
|  | 51592 | Zn-finger, RING |
|  | 51612 | Allergen V5/Tpx-1 related |
|  | 51613 | BTB/POZ |
|  | 51638 | Amidase |
|  | 51659 ^b^ | Tyrosine protein kinase, active site |
|  | 51681 | 3'-5' exonuclease |
|  | 78038 | C4-dicarboxylate transporter/malic acid transport protein |
|  | 88520 | Bile acid:sodium symporter |
|  | 88521 | Rhodanese-like |
|  | 97533 | Zn-finger, C2H2 type |
|  | 97547 ^b^ | ATP-dependent DNA ligase |
|  | 97549 | BPD_TRANSP_INN_MEMBR |
|  | 97573 ^b^ | Transcription factor, MADS-box |
|  | 97575 | Tyrosine protein kinase, active site |
|  | 97582 | Cof protein |
|  | 97584 ^b^ | Cyclin-like F-box |
|  | 97585 | Fungal transcriptional regulatory protein, N-terminal |
|  | 97592 | Fungal transcriptional regulatory protein, N-terminal |
|  | 97613 | Cytochrome c heme-binding site |
|  | 101931 | Camphor resistance CrcB protein |
|  | 106589 ^b^ | Forkhead-associated |
|  | 111731 | N-6 Adenine-specific DNA methylase |
|  | 111740 | ATP-dependent helicase, DEAD-box |
| Chromosome 15 | 51695 | Beta tubulin |
|  | 97626 | Alpha tubulin |
|  | 97640 | CRYSTALLIN_BETAGAMMA |
|  | 97646 | Cupin region |
|  | 97668 ^b^ | Helix-turn-helix, Fis-type |
|  | 97675 | Ferritin/ribonucleotide reductase-like |
| Chromosome 16 | 97702 | Peptidase A4, scytalidopepsin B |
|  | 97707 | R3H domain |
|  | 97717 | Peptidase S8 and S53, subtilisin, kexin, sedolisin |
|  | 97741 | dsRNA-binding domain-like |
|  | 106635 ^b^ | Homeobox |
| Chromosome 17 | 51731 | Prefoldin |
|  | 51740 | Heat shock protein Hsp20 |
|  | 97780 | Peptidase C48, SUMO/Sentrin/Ubl1 |
|  | 97790 ^b^ | Glycoside hydrolase, family 11 |
|  | 97837 | Shugoshin, N terminal |
|  | 106639 | Zn-finger, C2H2 type |
| Chromosome 18 | 19703 | HAT dimerisation |
|  | 30482 | Serine/threonine protein kinase |
|  | 51763 ^b^ | DEAD/DEAH box helicase, N-terminal |
|  | 97851 ^b^ | Ankyrin |
|  | 97867 ^b^ | TonB box, N-terminal |
|  | 97908 | DHH phosphoesterase |
|  | 111781 ^b^ | Calcium-binding EF-hand |
| Chromosome 19 | 27948 | BTB/POZ |
|  | 31017 | Chaperonin Cpn60/TCP-1 |
|  | 97915 ^b^ | Myb, DNA-binding |
|  | 97929 | Carbonic anhydrase, prokaryotic and plant |
|  | 97931 ^b^ | Thaumatin, pathogenesis-related |
|  | 97943 | H+-transporting two-sector ATPase, alpha/beta subunit, central region |
|  | 97953 ^b^ | Eukaryotic RNA polymerase II heptapeptide repeat |
|  | 111792 | Arthropod hemocyanin/insect LSP |
| Chromosome 20 | 98020 ^b^ | TonB box, N-terminal |
|  | 98042 | Helix-turn-helix, Fis-type |
|  | 98050 ^b^ | Inorganic pyrophosphatase |
|  | 111795 | Cytochrome c heme-binding site |
| Chromosome 21 | 51798 | Tubulin/FtsZ, GTPase |
|  | 98073 | Asp/Glu racemase |
|  | 98102^b^ | Regulator of chromosome condensation, RCC1 |
|  | 98110 | Kinesin, motor region |

^a^ Protein IDs are from the *M. graminicola* gene catalog in the database of the Joint Genome Institute. The genome sequence and annotations are available from the JGI web portal at <http://www.jgi.doe.gov/Mgraminicola>.

^b^ These genes are unique to the dispensable chromosomes.
