# Supplementary material for: Finished Genome of the Fungal Wheat Pathogen Mycosphaerella graminicola Reveals Dispensome Structure, Chromosome Plasticity, and Stealth Pathogenesis
Source: PLoS Genet. 2011 Jun 9;7(6):e1002070. doi: 10.1371/journal.pgen.1002070 (PMC3111534; doi:10.1371/journal.pgen.1002070)
Supplement: Table S2 — Analysis of small RNA sequences (generated on the Illumina platform) for the presence of computationally predicted pre-microRNA-like (milRNA) sequences in germinated spores of Mycosphaerella graminicola isolate IPO323. (DOCX) [file pgen.1002070.s016.docx]

**Table S2.** Analysis of small RNA sequences (generated on the Illumina platform) for the presence of computationally predicted pre-microRNA-like (milRNA) sequences in germinated spores of *Mycosphaerella graminicola* isolate IPO323.

| Small-RNA library^a^ | Trimmed reads^b^ | Average length (nt) | Number^c^ | | | |
| --- | --- | --- | --- | --- | --- | --- |
|  |  |  | >1 | >10 | >100 | >1000 |
| Germinated spores | 5.926.175 | 26.1 | 220 | 65 | 26 | 2^d^ |

^a^ RNA was isolated with standard procedures and the small RNAs were isolated with the Illumina small RNA kit and sequenced using standard procedures.

^b^ The number remaining after quality control and removal of adapter sequences.

^c^ The total number of the 385 predicted non-redundant pre-milRNA sequences with the indicated number of perfect hits (i.e., no mismatches were allowed over the full length of the RNA read) in the RNA data set using Blast.

^d^ The two predicted pre-milRNA sequences with more than a thousand perfect hits in the small-RNA data occurred 7342 and 4134 times.
