# Supplementary material for: Finished Genome of the Fungal Wheat Pathogen Mycosphaerella graminicola Reveals Dispensome Structure, Chromosome Plasticity, and Stealth Pathogenesis
Source: PLoS Genet. 2011 Jun 9;7(6):e1002070. doi: 10.1371/journal.pgen.1002070 (PMC3111534; doi:10.1371/journal.pgen.1002070)
Supplement: Table S3 — Best non-self BLAST hits for 654 called genes on dispensable chromosomes of Mycosphaerella graminicola queried with tblastn against a combined database containing the GenBank nt and EST datasets plus M. graminicola version 2.0 and M. fijiensis v1.0 from the Joint Genome Institute. (DOCX) [file pgen.1002070.s017.docx]

**Table S3.** Best non-self BLAST hits (E = 1 × 10^-5^) for 654 called genes on dispensable chromosomes of *Mycosphaerella graminicola* queried with *tblastn* against a combined database containing the GenBank nt and EST datasets plus *M. graminicola* version 2.0 and *M. fijiensis* v1.0 from the Joint Genome Institute.

| Genus or type | Class | Order | Number |
| --- | --- | --- | --- |
| No hits | Not applicable | Not applicable | 225 |
| *M. graminicola* GenBank | Dothideomycetes | Capnodiales | 140 |
| *M. graminicola* JGI D chromosome | Dothideomycetes | Capnodiales | 136 |
| *M. graminicola* JGI core chromosome | Dothideomycetes | Capnodiales | 86 |
| *M. fijiensis* | Dothideomycetes | Capnodiales | 13 |
| Animal | Not applicable | Not applicable | 8 |
| Ajellomyces | Eurotiomycetes | Onygenales | 7 |
| Aspergillus | Eurotiomycetes | Eurotiales | 6 |
| Penicillium | Eurotiomycetes | Eurotiales | 5 |
| Phaeosphaeria | Dothideomycetes | Pleosporales | 5 |
| Cercospora | Dothideomycetes | Capnodiales | 3 |
| Coccidioides | Eurotiomycetes | Onygenales | 3 |
| Geomyces | Eurotiomycetes | Onygenales | 2 |
| Pyrenophora | Dothideomycetes | Pleosporales | 2 |
| Botryotinia | Leotiomycetes | Helotiales | 2 |
| Talaromyces | Eurotiomycetes | Eurotiales | 1 |
| Trichoderma | Sordariomycetes | Hypocreales | 1 |
| Uncinocarpus | Eurotiomycetes | Onygenales | 1 |
| Grosmannia | Sordariomycetes | Ophiostomatales | 1 |
| Neurospora | Sordariomycetes | Sordariales | 1 |
| Cordyceps | Sordariomycetes | Hypocreales | 1 |
| Pichia | Saccharomycetes | Saccharomycetales | 1 |
| Claviceps | Sordariomycetes | Hypocreales | 1 |
| Neosartorya | Eurotiomycetes | Eurotiales | 1 |
| Podospora | Sordariomycetes | Sordariales | 1 |
| Candida | Saccharomycetes | Saccharomycetales | 1 |
| Sclerotinia | Leotiomycetes | Helotiales | 1 |
| Chaetomium | Sordariomycetes | Sordariales | 1 |
