# Supplementary material for: Finished Genome of the Fungal Wheat Pathogen Mycosphaerella graminicola Reveals Dispensome Structure, Chromosome Plasticity, and Stealth Pathogenesis
Source: PLoS Genet. 2011 Jun 9;7(6):e1002070. doi: 10.1371/journal.pgen.1002070 (PMC3111534; doi:10.1371/journal.pgen.1002070)
Supplement: Table S4 — Numbers of predicted enzymes degrading hemicellulose, pectin and cutin across seven ascomycete species with sequenced genomes. (DOCX) [file pgen.1002070.s018.docx]

**Table S4.** Numbers of predicted enzymes degrading hemicellulose, pectin and cutin across seven ascomycete species with sequenced genomes.

|  | Saprophytes^a^ | | |  | Pathogens^a^ | | | | Plant cell |
| --- | --- | --- | --- | --- | --- | --- | --- | --- | --- |
| CAZy family^b^ | An | Nc | Tr |  | Fg | Mg | Mo | Sn | wall target |
| GH5 mannosidases^c^ | 6 | 1 | 1 |  | 2 | 0 | 2 | 2 | Hemicellulose |
| GH26 | 3 | 1 | 0 |  | 0 | 0 | 0 | 0 | Hemicellulose |
| GH62 | 2 | 0 | 1 |  | 1 | 1 | 3 | 3 | Hemicellulose |
| GH67 | 1 | 1 | 1 |  | 1 | 0 | 1 | 1 | Hemicellulose |
| GH10 | 3 | 4 | 1 |  | 5 | 2 | 5 | 7 | Hemicellulose |
| GH11 | 2 | 2 | 4 |  | 3 | 1 | 5 | 7 | Hemicellulose |
| GH115 | 1 | 1 | 1 |  | 2 | 1 | 0 | 2 | Hemicellulose |
| Total hemicellulases | 18 | 10 | 9 |  | 14 | 5 | 16 | 22 | Hemicellulose |
|  |  |  |  |  |  |  |  |  |  |
| GH28 | 9 | 2 | 4 |  | 6 | 2 | 3 | 4 | Pectin |
| GH78 | 8 | 0 | 1 |  | 7 | 2 | 1 | 4 | Pectin |
| GH88 | 2 | 0 | 0 |  | 1 | 0 | 1 | 1 | Pectin |
| GH105 | 3 | 1 | 1 |  | 3 | 2 | 3 | 3 | Pectin |
| PL1 | 8 | 1 | 0 |  | 9 | 2 | 2 | 4 | Pectin |
| PL3 | 5 | 1 | 0 |  | 7 | 1 | 1 | 2 | Pectin |
| PL4 | 4 | 1 | 0 |  | 3 | 0 | 1 | 4 | Pectin |
| PL9 | 0 | 0 | 0 |  | 1 | 0 | 0 | 0 | Pectin |
| PL11 | 1 | 0 | 0 |  | 0 | 0 | 0 | 0 | Pectin |
| CE8 | 3 | 1 | 0 |  | 6 | 1 | 1 | 6 | Pectin |
| CE12 | 2 | 1 | 0 |  | 3 | 0 | 2 | 3 | Pectin |
| Total pectinases | 45 | 8 | 6 |  | 46 | 10 | 15 | 31 | Pectin |
|  |  |  |  |  |  |  |  |  |  |
| GH43 | 15 | 7 | 2 |  | 17 | 10 | 19 | 15 | Pectin & hemicellulose |
| GH51 | 2 | 1 | 0 |  | 2 | 3 | 3 | 2 | Pectin & hemicellulose |
| GH53 | 1 | 1 | 0 |  | 1 | 2 | 1 | 1 | Pectin & hemicellulose |
| GH54 | 1 | 1 | 2 |  | 1 | 1 | 1 | 1 | Pectin & hemicellulose |
| GH93 | 2 | 2 | 0 |  | 2 | 1 | 1 | 3 | Pectin & hemicellulose |
| Total pectinases & hemicellulases | 21 | 12 | 4 |  | 23 | 17 | 25 | 22 | Pectin & hemicellulose |
|  |  |  |  |  |  |  |  |  |  |
| CE5 | 4 | 3 | 4 |  | 12 | 6 | 17 | 11 | Cutin |
|  |  |  |  |  |  |  |  |  |  |
| Overall total including cellulases (Table 3) | 117 | 78 | 50 |  | 133 | 43 | 128 | 148 | All cell wall substrates |

^a^ Species analyzed included the saprophytes *Aspergillus nidulan*s (An), *Neurospora crassa* (Nc), and *Trichoderma reesii* (Tr), and the plant pathogens *Fusarium graminearum* (Fg), *Mycosphaerella graminicola* (Mg), *Magnaporthe oryzae* (Mo), and *Stagonospora nodorum* (Sn).

^b^ Families defined in the Carbohydrate-active enzymes database (www.cazy.org; [26]).

^c^ GH5 is a family containing many different enzyme activities; only those targeting the stated substrate are included.
