# Supplementary material for: Finished Genome of the Fungal Wheat Pathogen Mycosphaerella graminicola Reveals Dispensome Structure, Chromosome Plasticity, and Stealth Pathogenesis
Source: PLoS Genet. 2011 Jun 9;7(6):e1002070. doi: 10.1371/journal.pgen.1002070 (PMC3111534; doi:10.1371/journal.pgen.1002070)
Supplement: Table S5 — Total numbers of predicted CAZymes in Mycosphaerella graminicola and selected ascomycetes. (DOCX) [file pgen.1002070.s019.docx]

**Table S5.** Total numbers of predicted CAZymes in *Mycosphaerella graminicola* and selected ascomycetes.

| Species | GH | GT | PL | CE | CBM | EXPN |
| --- | --- | --- | --- | --- | --- | --- |
| *Mycosphaerella graminicola* | 184 | 97 | 3 | 20 | 20 | 3 |
| *Trichoderma reesei* | 192 | 93 | 6 | 17 | 48 | 4 |
| *Fusarium graminearum* | 247 | 102 | 21 | 44 | 67 | 4 |
| *Neurospora crassa* | 173 | 76 | 4 | 22 | 42 | 1 |
| *Magnaporthe grisea* | 232 | 92 | 5 | 47 | 65 | 1 |
| *Aspergillus nidulans* | 251 | 91 | 21 | 31 | 41 | 1 |
| *Stagonospora nodorum* | 284 | 92 | 10 | 57 | 74 | 4 |

GH = glycoside hydrolases; GT = glycosyl transferases; PL = polysaccharide lyases; CE =carbohydrate esterases; CBM = carbohydrate binding modules; EXPN = distantly related to plant expansins.

Predicted CAZymes were identified using the carbohydrate-active enzymes database tools ([www.cazy.org](http://www.cazy.org/)).
