# Supplementary material for: Finished Genome of the Fungal Wheat Pathogen Mycosphaerella graminicola Reveals Dispensome Structure, Chromosome Plasticity, and Stealth Pathogenesis
Source: PLoS Genet. 2011 Jun 9;7(6):e1002070. doi: 10.1371/journal.pgen.1002070 (PMC3111534; doi:10.1371/journal.pgen.1002070)
Supplement: Table S6 — PFAM domains that are expanded in the genome of Mycosphaerella graminicola relative to those of five other Ascomycetesa and two plant-pathogenic Stramenopilesb. (DOCX) [file pgen.1002070.s020.docx]

**Table S6.** PFAM domains that are expanded in the genome of *Mycosphaerella graminicola* relative to those of five other Ascomycetes^a^ and two plant-pathogenic Stramenopiles^b^.

| Name | Mgram | Snod | Fgram | Moryz | Trees | Ncra | Pram | Psoj | Domain function |
| --- | --- | --- | --- | --- | --- | --- | --- | --- | --- |
| PF01432 | 16 | 2 | 4 | 2 | 2 | 2 | 3 | 3 | Peptidase_M3 |
| PF00128 | 13 | 5 | 7 | 9 | 4 | 9 | 2 | 1 | Alpha-amylase |
| PF01070 | 12 | 8 | 10 | 6 | 6 | 3 | 2 | 1 | FMN_dh |
| PF05577 | 10 | 4 | 2 | 6 | 3 | 2 | 5 | 5 | Peptidase_S28 |
| PF09286 | 10 | 6 | 3 | 5 | 5 | 3 | 2 | 2 | Pro-kuma_activ |
| PF00180 | 9 | 7 | 8 | 7 | 7 | 5 | 3 | 2 | Iso_dh |
| PF03061 | 9 | 4 | 5 | 6 | 6 | 5 | 4 | 3 | 4HBT |
| PF04193 | 8 | 7 | 7 | 5 | 5 | 6 | 4 | 1 | PQ-loop |
| PF00320 | 8 | 6 | 7 | 7 | 6 | 6 | 0 | 0 | GATA |
| PF02714 | 7 | 6 | 4 | 4 | 5 | 5 | 2 | 3 | DUF221 |
| PF00682 | 6 | 4 | 4 | 5 | 4 | 4 | 3 | 3 | HMGL-like |
| PF06747 | 6 | 5 | 3 | 3 | 3 | 4 | 4 | 2 | CHCH |
| PF00250 | 6 | 4 | 4 | 3 | 4 | 3 | 0 | 0 | Fork_head |
| PF01501 | 6 | 5 | 5 | 2 | 2 | 2 | 1 | 1 | Glyco_transf_8 |
| PF00206 | 5 | 4 | 3 | 3 | 3 | 3 | 3 | 3 | Lyase_1 |
| PF00130 | 5 | 2 | 3 | 2 | 2 | 2 | 2 | 2 | C1_1 |
| PF03476 | 4 | 2 | 3 | 2 | 2 | 2 | 2 | 2 | MOSC_N |
| PF00686 | 4 | 3 | 2 | 3 | 1 | 2 | 0 | 0 | CBM_20 |
| PF03055 | 4 | 3 | 2 | 3 | 1 | 1 | 0 | 0 | RPE65 |
| PF05875 | 3 | 2 | 2 | 1 | 2 | 1 | 2 | 1 | aPHC |
| PF04303 | 3 | 2 | 2 | 2 | 1 | 0 | 0 | 0 | DUF453 |
| PF01645 | 3 | 1 | 1 | 1 | 1 | 1 | 1 | 1 | Glu_synthase |
| PF08760 | 3 | 2 | 0 | 2 | 1 | 2 | 0 | 0 | DUF1793 |
| PF06964 | 3 | 1 | 2 | 2 | 0 | 1 | 0 | 0 | Alpha-L-AF_C |
| PF08323 | 3 | 0 | 0 | 1 | 0 | 2 | 0 | 0 | Glyco_transf_5 |
| PF09260 | 3 | 0 | 0 | 1 | 0 | 2 | 0 | 0 | DUF1966 |
| PF00692 | 2 | 1 | 1 | 1 | 1 | 1 | 1 | 1 | dUTPase |
| PF01916 | 2 | 1 | 1 | 1 | 1 | 1 | 1 | 1 | DS |
| PF01715 | 2 | 1 | 1 | 1 | 1 | 1 | 1 | 1 | IPPT |
| PF08313 | 2 | 1 | 1 | 1 | 1 | 1 | 0 | 0 | SCA7 |
| PF03641 | 2 | 1 | 1 | 1 | 1 | 1 | 0 | 0 | Lysine_decarbox |
| PF03600 | 2 | 1 | 1 | 1 | 1 | 1 | 0 | 0 | CitMHS |
| PF02705 | 2 | 1 | 1 | 1 | 0 | 1 | 0 | 0 | K_trans |
| PF07558 | 2 | 1 | 1 | 0 | 1 | 1 | 0 | 0 | Shugoshin_N |
| PF09296 | 2 | 1 | 1 | 0 | 1 | 1 | 0 | 0 | NUDIX-like |
| PF02982 | 2 | 1 | 1 | 1 | 0 | 1 | 0 | 0 | Scytalone_dh |
| PF01422 | 2 | 0 | 1 | 0 | 1 | 1 | 0 | 0 | zf-NF-X1 |
| PF05390 | 2 | 1 | 0 | 0 | 0 | 0 | 0 | 0 | KRE9 |

^a^ Species abbreviations for Ascomycetes: Mgram, *M. graminicola*; Snod, *Stagonospora nodorum*; Fgram, *Fusarium graminearum*; Moryz, *Magnaporthe oryzae*; Trees, *Trichoderma reesei*; and Ncra, *Neurospora crassa*.

^b^ Species abbreviations for Stramenopiles: Pram, *Phytophthora ramorum*; Psoj, *P. sojae*.
