# Supplementary material for: Finished Genome of the Fungal Wheat Pathogen Mycosphaerella graminicola Reveals Dispensome Structure, Chromosome Plasticity, and Stealth Pathogenesis
Source: PLoS Genet. 2011 Jun 9;7(6):e1002070. doi: 10.1371/journal.pgen.1002070 (PMC3111534; doi:10.1371/journal.pgen.1002070)
Supplement: Table S7 — PFAM domains that are expanded in the genome of Mycosphaerella graminicola relative to those of five other Ascomycetesa but not the two plant-pathogenic Stramenopilesb. (DOCX) [file pgen.1002070.s021.docx]

**Table S7.** PFAM domains that are expanded in the genome of *Mycosphaerella graminicola* relative to those of five other Ascomycetes^a^ but not the two plant-pathogenic Stramenopiles^b^.

| Name | Mgram | Snod | Fgram | Moryz | Trees | Ncra | Pram | Psoj | Domain function |
| --- | --- | --- | --- | --- | --- | --- | --- | --- | --- |
| PF00560 | 16 | 14 | 14 | 12 | 14 | 15 | 84 | 100 | LRR_1 |
| PF07728 | 16 | 13 | 12 | 13 | 11 | 12 | 33 | 34 | AAA_5 |
| PF00043 | 14 | 10 | 7 | 4 | 9 | 4 | 12 | 19 | GST_C |
| PF00450 | 13 | 10 | 9 | 6 | 5 | 4 | 14 | 16 | Peptidase_S10 |
| PF00230 | 10 | 8 | 5 | 6 | 7 | 1 | 29 | 32 | MIP |
| PF00557 | 10 | 9 | 9 | 8 | 9 | 7 | 11 | 11 | Peptidase_M24 |
| PF04055 | 9 | 8 | 7 | 5 | 7 | 5 | 12 | 12 | Radical_SAM |
| PF00782 | 8 | 7 | 6 | 5 | 6 | 5 | 17 | 23 | DSPc |
| PF01553 | 8 | 6 | 5 | 6 | 5 | 5 | 12 | 10 | Acyltransferase |
| PF01590 | 6 | 4 | 3 | 4 | 3 | 4 | 31 | 32 | GAF |
| PF01757 | 6 | 2 | 1 | 2 | 1 | 2 | 10 | 14 | Acyl_transf_3 |
| PF00520 | 4 | 2 | 2 | 2 | 3 | 2 | 40 | 61 | Ion_trans |
| PF03330 | 4 | 1 | 2 | 2 | 2 | 1 | 13 | 12 | DPBB_1 |

^a^ Species abbreviations for Ascomycetes: Mgram, *M. graminicola*; Snod, *Stagonospora nodorum*; Fgram, *Fusarium graminearum*; Moryz, *Magnaporthe oryzae*; Trees, *Trichoderma reesei*; and Ncra, *Neurospora crassa*.

^b^ Species abbreviations for Stramenopiles: Pram, *Phytophthora ramorum*; Psoj, *P. sojae*.
