# Supplementary material for: Finished Genome of the Fungal Wheat Pathogen Mycosphaerella graminicola Reveals Dispensome Structure, Chromosome Plasticity, and Stealth Pathogenesis
Source: PLoS Genet. 2011 Jun 9;7(6):e1002070. doi: 10.1371/journal.pgen.1002070 (PMC3111534; doi:10.1371/journal.pgen.1002070)
Supplement: Table S8 — Assembly statistics for the Mycosphaerella graminicola version 1 (8.9× draft) and version 2 (finished) sequences compared to the 10× draft sequence of Stagonospora nodorum. (DOCX) [file pgen.1002070.s022.docx]

**Table S8.** Assembly statistics for the *Mycosphaerella graminicola* version 1 (8.9× draft) and version 2 (finished) sequences compared to the 10× draft sequence of *Stagonospora nodorum.*

| Category | *M. graminicola* v1.0 (draft) | *M. graminicola* v2.0 (finished) | *S .nodorum*  draft |
| --- | --- | --- | --- |
| Sequence total, Mb | 41.2 | 39.7 | 37.1 |
| Number of scaffolds | 129 | 21 | 107 |
| Number of contigs | 1,008 | 21 | 496 |
| Scaffold N50/L50 | 6/2.4 Mb | Finished | 13/1.1 Mb |
| Coverage | 8.88× | Finished | > 10× |
| Gaps, Mbp | 2.5 (7.5%) | 0.006 (0.01%) | 0.16 (0.43%) |
| Repeats, Mbp |  | 7.17 | 2.62 |
| Repeats, percent |  | 18 | 7 |
